# Photoreceptor oxidative stress in hyperoxia-induced proliferative retinopathy accelerates *rd8* degeneration

**DOI:** 10.1371/journal.pone.0180384

**Published:** 2017-07-03

**Authors:** Michelle Lajko, Herminio J. Cardona, Joann M. Taylor, Kathryn N. Farrow, Amani A. Fawzi

**Affiliations:** 1 Department of Ophthalmology, Feinberg School of Medicine at Northwestern University, Chicago, Illinois, United States of America; 2 Department of Pediatrics, Feinberg School of Medicine at Northwestern University, Chicago, Illinois, United States of America; University of Florida, UNITED STATES

## Abstract

To investigate the impact of photoreceptor oxidative stress on photoreceptor degeneration in mice carrying the *rd8* mutation (C57BL/6N). We compared the hyperoxia-induced proliferative retinopathy (HIPR) model in two mouse strains (C57BL/6J and C57BL/6N). Pups were exposed to 75% oxygen, starting at birth and continuing for 14 days (P14). Mice were euthanized at P14, or allowed to recover in room air for one day (P15), seven days (P21), or 14 days (P28). We quantified retinal thickness and the length of residual photoreceptors not affected by rosette formation. In addition we explored differences in retinal immunostaining for NADPH oxidase 4 (NOX4), Rac1, vascular endothelium, and activated Mϋller cells. We analyzed photoreceptor oxidative stress using DCF staining in cross sections and quantified NOX4 protein levels using western blotting. C57BL/6N mice in HIPR showed increased oxidative stress, NOX4, and Rac1 in the photoreceptors at P14 and P15 compared to C57BL/6J. In addition, we observed significant progression of photoreceptor degeneration, with significantly accelerated rosette formation in C57BL/6N under HIPR, compared to their room air counterparts. Furthermore, C57BL/6N under HIPR had significantly thinner central retinas than C57BL/6J in HIPR. We did not find a difference in vascular disruption or Mϋller cell activation comparing the two strains in hyperoxia. In HIPR, the C57BL/6N strain carrying the *rd8* mutation showed significantly accelerated photoreceptor degeneration, mediated via exacerbated photoreceptor oxidative stress, which we believe relates to Rac1-NOX dysregulation in the setting of Crb1 loss-of-function.

## Introduction

The mutation known as retinal degeneration 8 (*rd8*) is caused by a spontaneous frameshift mutation resulting in a premature truncation in crumbs 1 (*Crb1*). On funduscopic examination, the *rd8* mice show large white spots in the nasal quadrant, progressively spreading along with a slow process of photoreceptor degeneration [1, 2]. Histologically, the *rd8* mutation is characterized by retinal folding, photoreceptor rosettes, outer nuclear layer loss, and retinal degeneration [1]. The photoreceptors are 25% shorter in mice with *rd8* at 28 days of age, progressing to disorganized outer segments, with shortened inner segments by 10 weeks of age [2]. At five months of age, the photoreceptors lose their distinct, stratified organization, with complete loss of the outer segments in 35-month-old mice [2].

The protein product of the *Crb1* gene is localized to the outer limiting membrane (OLM), which is comprised of highly specialized junctions that maintain retinal integrity and polarity. One of these junctions is the subapical region (SAR), where the photoreceptor inner segments and Mϋller glial cells connect. Crb1 localizes at the SAR on the microvilli of Mϋller glial cells [3–5]. Loss of Crb1 function disrupts the photoreceptor-Mϋller cell adhesion, which in turn weakens the integrity of the OLM, ultimately contributing to photoreceptor loss and the appearance of the classic rosettes of involuted photoreceptors [2–5].

In *Drosophila*, crb has been shown to control epithelial polarity by counteracting phosphoinositol 3-kinase (PI3K) and Rac1, a Rho GTPase[6–10]. Rac1 is important in cell-cell interaction, cell polarity, and cell migration [11]. Crb represses PI3K and Rac1 to stabilize the OLM integrity. It acts as a negative regulator of the Rac-NADPH oxidase (NOX)-dependent production of reactive oxygen species (ROS) in photoreceptors [9]. In a *Drosophila* model, loss of crb was shown to lead to dysregulation of the Rac1-NOX pathway, causing superoxide overproduction, and destruction of the photoreceptors [9]. Similarly, mice with constitutively active photoreceptor Rac1 have been shown to exhibit an increase in NOX-meditated oxidative stress that leads to photoreceptor degeneration [12]. Histologically, these mice had outer nuclear layer folds, with loss of photoreceptor orientation, a phenotype that simulates the *rd8* mutation [12, 13].

Given the importance of Crb1 in regulating Rac1 and limiting the production of photoreceptor superoxide, we sought to explore the effect of neonatal hyperoxia and neonatal proliferative retinopathy on mice carrying the *rd8* mutation. We used the hyperoxia-induced proliferative (HIPR) model [14], where new-born pups are placed in high oxygen (75%) for 14 days. In the current study, we compared the effects of HIPR in two mouse strains: C57BL/6J (B6J) and C57BL/6N (B6N). These two strains were chosen since the C57BL/6N were homozygous for the *rd8* mutation, while the C57BL/6J carried the wild-type *Crb1* [15, 16]. We hypothesized that neonatal oxidative stress would exacerbate the *rd8* phenotype characterized by rosette formation in B6N mice via Rac1 dysregulation. We found that C57BL/6N develop an accelerated retinal degeneration, with loss of about 80% of photoreceptors within the first 4 weeks, compared to their room air raised littermates. These findings demonstrate that Crb1 is important in regulating photoreceptor oxidative stress and may have important translational implications to human disease, particularly infants carrying *CRB1* mutations.

## Methods

### Hyperoxia-induced proliferative retinopathy model

This study was approved by the Institutional Animal Care and Use Committee at Northwestern University and all animal procedures were performed in compliance with the Institutional Animal Care and Use Committee at Northwestern University and the Guide for the Care and Use of Laboratory Animals of the National Institutes of Health. Mice were sacrificed with isoflurane overdose and cervical dislocation.

**Fig 1:**
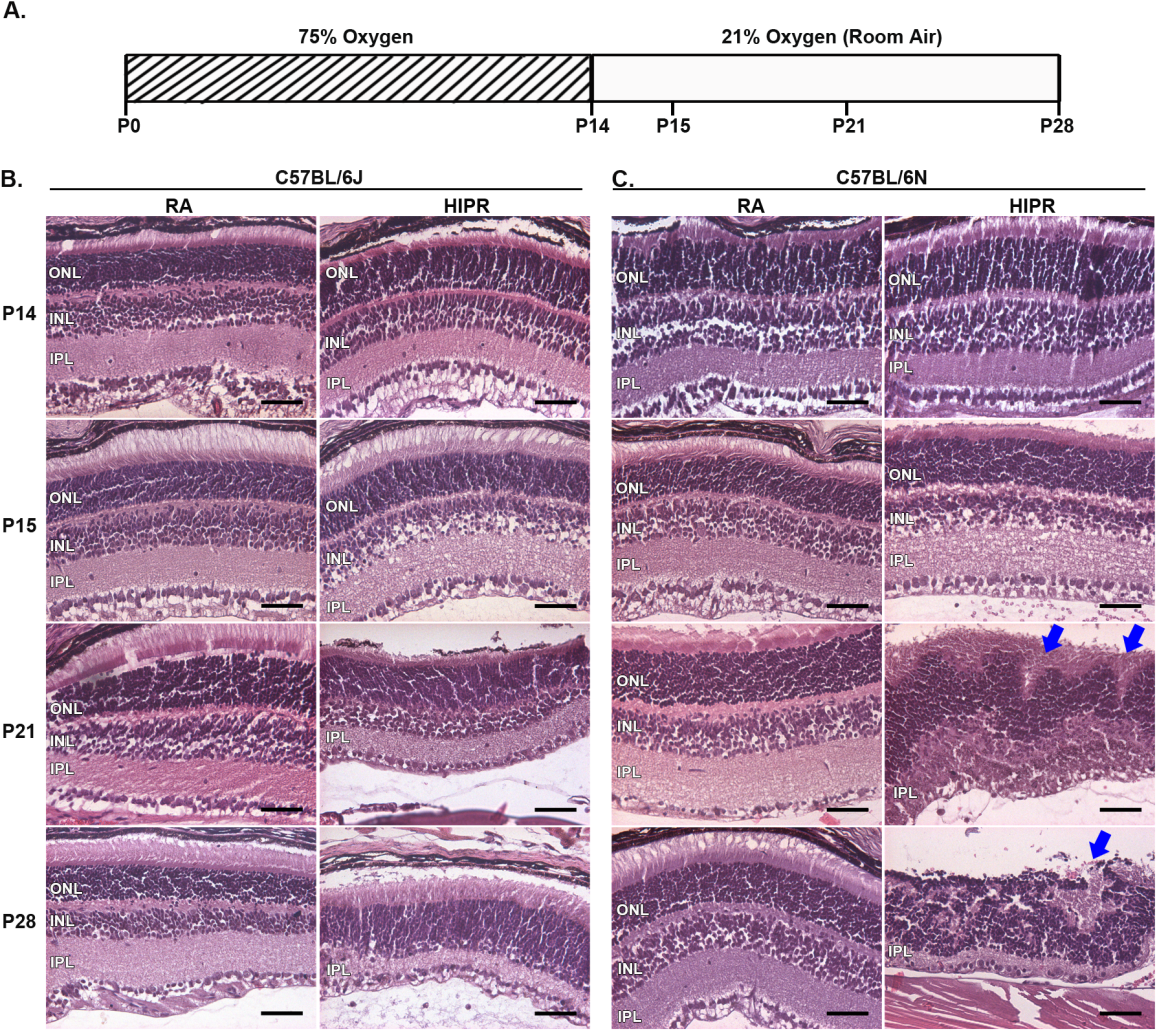
Retinal folding and loss of photoreceptors in C57BL/6N compared to C57BL/6J in hyperoxia-induced proliferative retinopathy (HIPR). (A) In the hyperoxia-induced proliferative retinopathy model, mice were exposed to hyperoxia for 14 days and either sacrificed or returned to room air for one day (P15), seven days (P21), or 14 days (P28). Room air (RA) control pups were sacrificed at the same time points. Retinal cross sections were stained with hematoxylin and eosin (B, C). Rosettes of photoreceptors were seen in C57BL/6N HIPR mice at P21 and more prominently at P28 (blue arrows). Scale bar, 50 μm (N = 3–5). ONL, outer nuclear layer. INL, inner nuclear layer. IPL, inner plexiform layer.

Two strains of mice, C57BL/6J (000664, Jackson Laboratory, Bar Harbor, ME) and C57BL/6N (027, Charles River, Wilmington, MA), were used. The C57BL/6N mice carry the *rd8* mutation while the C57BL/6J mice carry the wild-type *Crb1* gene [15, 16]. We have recently characterized the retinopathy in the HIPR model [14, 17, 18]. Briefly, newborn pups are placed in a Plexiglas chamber with an oxygen controller (Pro-Ox 110; Biospherix, Lacona, NY) and exposed to 75% oxygen from birth to P14. Dams were rotated from hyperoxia to room air every 24 hours to avoid oxygen toxicity [18]. Mice were either sacrificed after two weeks in hyperoxia (P14) or removed to room air for one day (P15), one week (P21), or two weeks (P28) and then sacrificed (Fig 1A). Control, age-matched pups were raised in room air. Both male and female mice were used in control and HIPR groups in equal numbers. The HIPR model was reproduced in 42 litters (with corresponding room air controls) over a period of three years. The data presented is collated from experiments with mice in different litters.

### Detection of *rd8* mutation by PCR

DNA was isolated from tail biopsies and DNA samples were amplified separately for the wildtype and mutant *rd8* alleles as previously described, using the mCrb1-mF1, mCrb1-mF2, and mCrb1-mR primers [2]. The PCR reaction was carried out in a 25 μL reaction volume containing 20ng DNA, 1.25 μM forward and reverse primer, and 2X Master Mix (Thermo Fisher Scientific, Waltham, MA). PCR reactions were performed using the following conditions: denaturation 94°C for 5 minutes, amplification for 40 cycles at 94°C 30 seconds, 65°C for 30 seconds, 72°C for 30 seconds, and final extension at 72°C for 7 minutes. The wildtype allele produces a 220 bp fragment and the mutant *rd8* allele produces a 244 bp fragment.

### Retinal thickness and residual photoreceptor analysis

Enucleated eyes were fixed in 10% neutral buffered formalin for 24 hours or fixed in 4% paraformaldehyde for four hours. Paraffin embedded eyes were sectioned (7 μm), deparaffinized, and hematoxylin and eosin stained. Images were taken with Nikon 80i Eclipse microscope (Nikon, Tokyo, Japan) using a Photometrics CoolSnap CF camera (Photometrics, Tucson, AZ).

Retinal thickness analysis was performed using ImageJ software (NIH, Bethesda, MD), as previously reported [19]. Thickness was measured by two masked observers at multiple locations: 100 μm on either side of the optic nerve and in the retinal periphery. The measurements were calculated using *distance_between_polylines*.*java* ImageJ plug-in [20]. This tool allows multiple thickness measurements to be automatically calculated and averaged. We calculated the retinal thickness as the average of three to four eyes per time point.

The percentage of residual healthy photoreceptors was determined by dividing the length of visible photoreceptors by the total retinal pigment epithelium length, per retinal cross-section. Two independent masked observers analyzed four to five separate cross-sections (40 μm apart) per eye. The measurements obtained by the two observers were compared to calculate the interclass correlation coefficient (ICC).

### Retinal flat mount analysis

Retinas were processed and stained as previously reported [21]. Eyes were fixed in 10% neutral buffered formalin for 24 hours, retinas were dissected and the vitreous removed. Retinal cups were permeabilized for 18 hours with phosphate buffered saline (PBS) supplemented with 0.1% Triton X-100. After blocking in 10% donkey serum, 1% bovine serum albumin (BSA), 0.1% Triton X-100 diluted in PBS for five hours, retinas were immunostained with GS-Isolectin Alexa Fluor 594 (IB4, 1:75 dilution, I21413, Thermo Fisher Scientific) for 18 hours. After several washes, retinas were flattened and mounted with ProLong Gold Antifade reagent (Thermo Fisher Scientific). Flat mounts were masked and quantified by two independent observers. Measurements were made using ImageJ (NIH) to quantify the avascular retinal area (in pixels) as a percentage of the total retinal area.

### Immunohistochemistry

Enucleated eyes were fixed in 4% paraformaldehyde, embedded in paraffin, sectioned (7 μm), deparaffinized, and underwent antigen retrieval in sodium citrate buffer (10 nM sodium citrate, 0.05% Tween-20, pH 6.0) at 80°C for 20 minutes NADPH oxidase 4 (NOX4). For Rac1, RhoA, and Cdc42, enucleated eyes were fresh frozen, sectioned (7 μm), and fixed for 15 minutes with 4% paraformaldehyde. Sections were blocked (10% donkey serum, 0.1% Triton X-100) for one hours and then incubated with primary antibody for 18 hours at 4°C. Primary antibodies used were rabbit anti-glial fibrillary acidic protein (GFAP, 1:200 dilution, ab7260, Abcam, Cambridge, UK), rabbit anti-NOX4 (1:200 dilution, 14347-1-AP, Proteintech, Rosemont, IL), mouse anti-Rac1 (1:200 dilution, 05–389, EMD Millipore, Billerica, MA), rabbit anti-RhoA (1:100 dilution, ab187027, Abcam), and rabbit anti-Cdc42 (1:100 dilution, ab64533, Abcam). After washes, the sections were incubated with donkey anti-rabbit FITC (1:100 dilution, ab97084, Abcam), IB4 (1:100 dilution, I21413, Thermo Fisher Scientific), donkey anti-mouse Alexa Fluor 647 (1:200 dilution, ab150107, Abcam), or donkey anti-rabbit Rhodamine Red (1:200 dilution, 711-295-152, Jackson Immunoresearch, West Grove, PA) for 1 hour. Rinsed sections were stained with 0.1% or 0.5% Sudan black diluted in 70% ethanol to quench autofluorescence and counterstained with 4’, 6-diamidino-2-phenylindole (DAPI, R37605, Thermo Fisher Scientific), rinsed, mounted, and sealed. Sections were imaged with Nikon A1R+ confocal laser microscope system.

### Reactive oxygen species detection and immunohistochemistry of the outer limiting membrane (CD44)

Reactive oxygen species (ROS) generation was examined by dichlorofluorescein staining.[22] The non-fluorescent indicator, 2’, 7’-dichlorodihydrofluorescein diacetate (H_2_DCFDA, Thermo Fisher Scientific), is converted to the highly fluorescent 2’, 7’-dichlorofluorescein (DCF) when acetate groups were cleaved by intracellular esterases and oxidation. DCF staining is a direct measure of ROS. Unfixed retinal cryosections (10 μm) were incubated with 10 μM H_2_DCFDA at 37°C in a dark, humidified chamber for one hour. Sections were imaged immediately with a Zeiss LSM-510 Meta confocal microscope (Zeiss, Oberkochen, Germany).

The sections were then stained for an adhesion receptor restricted to the Mϋller glial cell end-feet, CD44 [2, 23]. Immediately after imaging for DCF, the coverslips were carefully removed and the sections were fixed for 10 minutes in -20°C acetone. Following air drying and several washes, endogenous hydrogen peroxide was blocked with 3% hydrogen peroxide. After blocking in 10% donkey serum, sections were incubated in rat anti-CD44 (1:100 dilution, 553131, BD Biosciences, San Jose, CA) and then incubated with biotin conjugated donkey anti-rat (1:200 dilution, 112-065-167, Jackson ImmunoResearch). The sections were incubated with streptavidin Alexa Fluor 594 conjugate (1:200 dilution, S-11227, Thermo Fisher Scientific) for one hour and counterstained with To-Pro3 (1:300 dilution, T3605, Thermo Fisher Scientific). Sections were imaged with the Zeiss LSM-510 Meta confocal microscope.

### Quantifying fluorescence intensity

We determined fluorescence intensity using three different cross-sections per eye (N = 3 eyes per group) using ImageJ (NIH) [24, 25]. The fluorescence was calculated using corrected total cell fluorescence (CTCF) using the following formula: CTCF = Integrated density–(Area of selection X Mean fluorescence of background areas). Reported data are mean ± standard error of mean (SEM).

### Western blotting

Western blotting was performed as previously described [26]. Immediately after enucleation, retinas were dissected and lysed in 1X Mg2+ lysis buffer (EMD Millipore) with a protease inhibitor (Sigma Aldrich, St. Louis, MO) and a phosphatase inhibitor (EMD Millipore). Samples were quantified with Bradford Reagent (Bio-Rad) [27]. 40 μg of total protein were separated on 4–20% Tris-glycine gel (Bio-Rad), transferred to nitrocellulose membrane (Bio-Rad), and blocked with 5% BSA diluted in Tris-buffered saline supplemented with Tween-20 (TBST) for one hour. Membranes were incubated with rabbit anti-NOX4 (1:200 dilution, sc-30141, Santa Cruz, Dallas, TX) for 18 hours or mouse anti-β-actin (1:5000 dilution, A5316, Sigma-Aldrich) diluted in 5% BSA in TBST for one hour. After washing, membranes were incubated with donkey anti-rabbit (1:5000 dilution, NA934, GE Healthcare, Piscataway, NJ) or horse anti-mouse IgG (1:5000 dilution, 7076, Cell Signaling, Boston, MA) and exposed with ECL Detection Reagents (GE Healthcare). Bands were analyzed using ChemiDoc XRS (Bio-Rad) and normalized to β-actin. Data are fold change ± SEM.

### Statistical analysis

All data were analyzed using SPSS (v.23.0; IBM Corp, Armonk, NY). Results are expressed as mean ± SEM. Mice from different litters (4–5 per group) were analyzed. Group differences were evaluated using ANOVA with Bonferroni’s *post hoc* comparisons. Results were considered statistically significant for p-value < 0.05.

## Results

### C57BL/6N in hyperoxia display significant progression of rosette formation compared to their room air counterparts, and significantly thinner retina than C57BL/6J mice in hyperoxia

**Fig 2:**
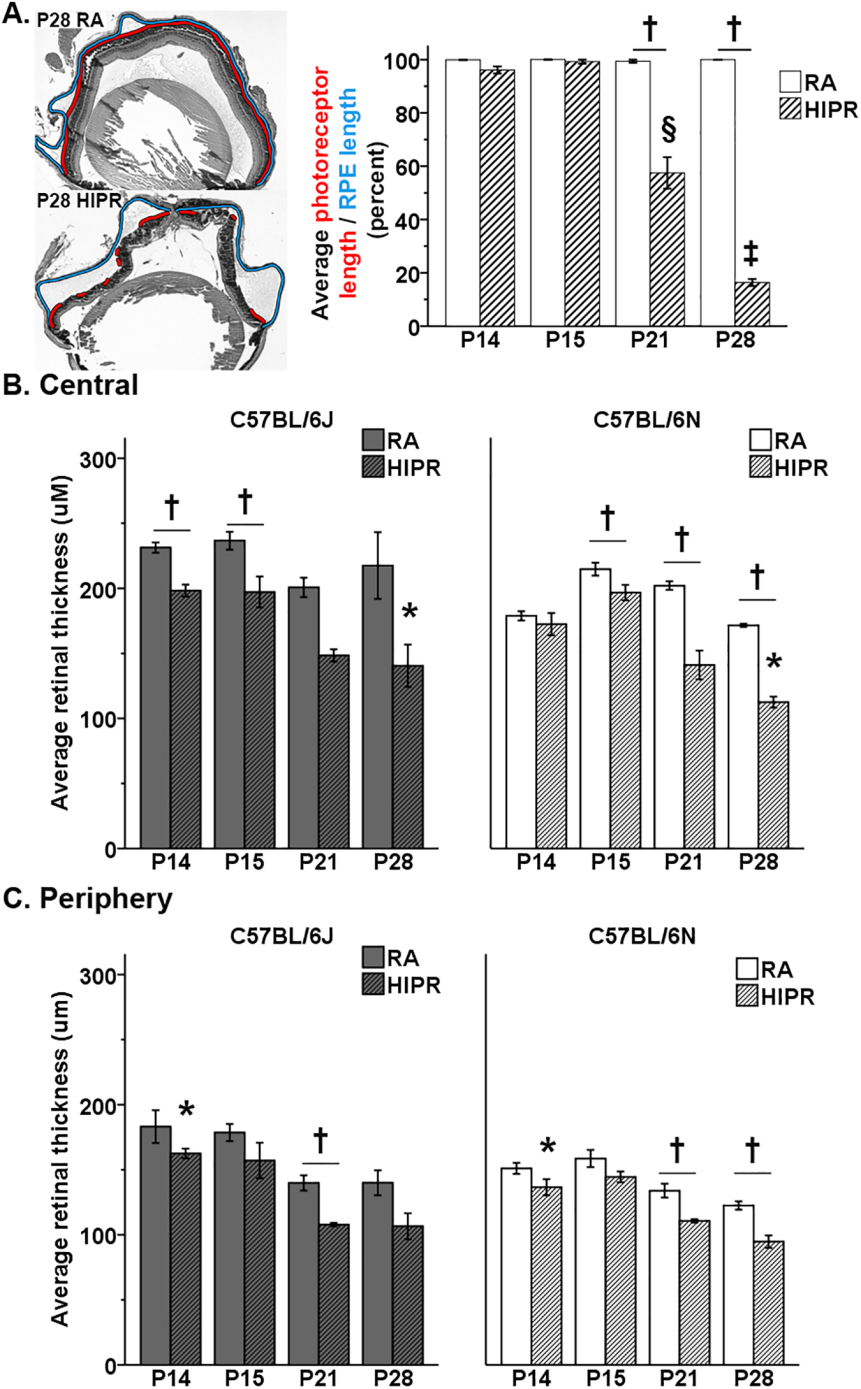
Loss of photoreceptors and retinal thinning are observed in the central and peripheral retina in both strains under hyperoxia-induced proliferative retinopathy (HIPR). (A) On the left, an example measurement of the length of intact photoreceptors (red) was normalized to the length of the retinal pigment epithelium (RPE, blue). Loss of photoreceptor orientation was only visible in C57BL/6N mice. (B, C) Retinal cross sections were measured at 100 μm on either sides of the optic nerve and in the retinal periphery. (B) Near the optic nerve, HIPR C57BL/6N had significantly thinner retinas at P15, P21, and P28 than their room air (RA) controls. (C) In the peripheral retina, HIPR C57BL/6N had significantly thinner retinas at P21 and P28 compared to room air controls. † p<0.05 HIPR to respective age-matched RA controls. § p<0.05 P21 HIPR C57BL/6N compared to P15 HIPR C57BL/6N. ‡ p<0.05 P28 HIPR C57BL/6N compared to P21 HIPR C57BL/6N. * p<0.05 HIPR C57BL/6J compared to HIPR C57BL/6N. Values were mean ± SEM (N = 3–4).

To identify morphological differences between C57BL/6J (B6J) and C57BL/6N (B6N) strains, histologic cross-sections were analyzed. In room air controls of both strains, we did not see any evidence of rosette formation at any time point (N = 3–4 per time point). The B6J in HIPR did not display the rosette formation and had continuous intact photoreceptors throughout the retina (Fig 1B), while the B6N HIPR mice revealed rosette structures involving the photoreceptors at P21 and P28 (Fig 1C, blue arrows and S1 Fig). In the B6N strain, the outer nuclear layer and inner nuclear layer were indistinct, precluding any measurements of the outer nuclear layer thickness or nuclei. Thus to quantify the loss of photoreceptors, the length of residual photoreceptors was normalized to the length of the retinal pigment epithelium (RPE) (Fig 2A, left). The ratio of intact photoreceptor (inner and outer segments) to RPE length decreased significantly in B6N HIPR retinas comparing P15 to P21 (99.2 ± 0.8%, ICC 0.98 vs 57.5 ± 5.9%, ICC 0.95, p<0.05) (Fig 2A), decreasing further from P21 to P28 (16.4 ± 1.3%, ICC 0.97, p<0.05) (Fig 2A). In the setting of HIPR, the retinal sublayers became largely disorganized and indistinguishable in *rd8* eyes (B6N) at P28 (Fig 1C).

The total retinal thickness was measured near the optic nerve and in the peripheral retina. In B6N mice, retinas were significantly thinner in HIPR than room air controls at P15, P21, and P28 near the optic nerve (Fig 2B) and only at P21 and P28 in the periphery (p<0.05, Fig 2C). In contrast, 6J HIPR retinas were significantly thinner than room air controls centrally at P14 and P15 (p<0.05, Fig 2B) and in the periphery at P21 (p<0.05, Fig 2C).

Comparing the two strains near the optic nerve, B6N HIPR had significantly thinner retinas than B6J HIPR at P14 (172.4 ± 8.6 μm vs 198.2 ± 4.6 μm, p<0.05) and P28 (112.5 ± 4.3 μm vs 140.5 ± 16.3 μm, p<0.05, Fig 2B). In the periphery, the only strain difference was seen at P14 where B6N HIPR showed significantly thinner retina than B6J HIPR (136.6 ± 6.2 μm vs 162.5 ± 3.8 μm, p<0.05, Fig 2C).

### Disorganized angiogenesis occurs in both C57BL/6J and C57BL/6N in hyperoxia-induced proliferative retinopathy

**Fig 3:**
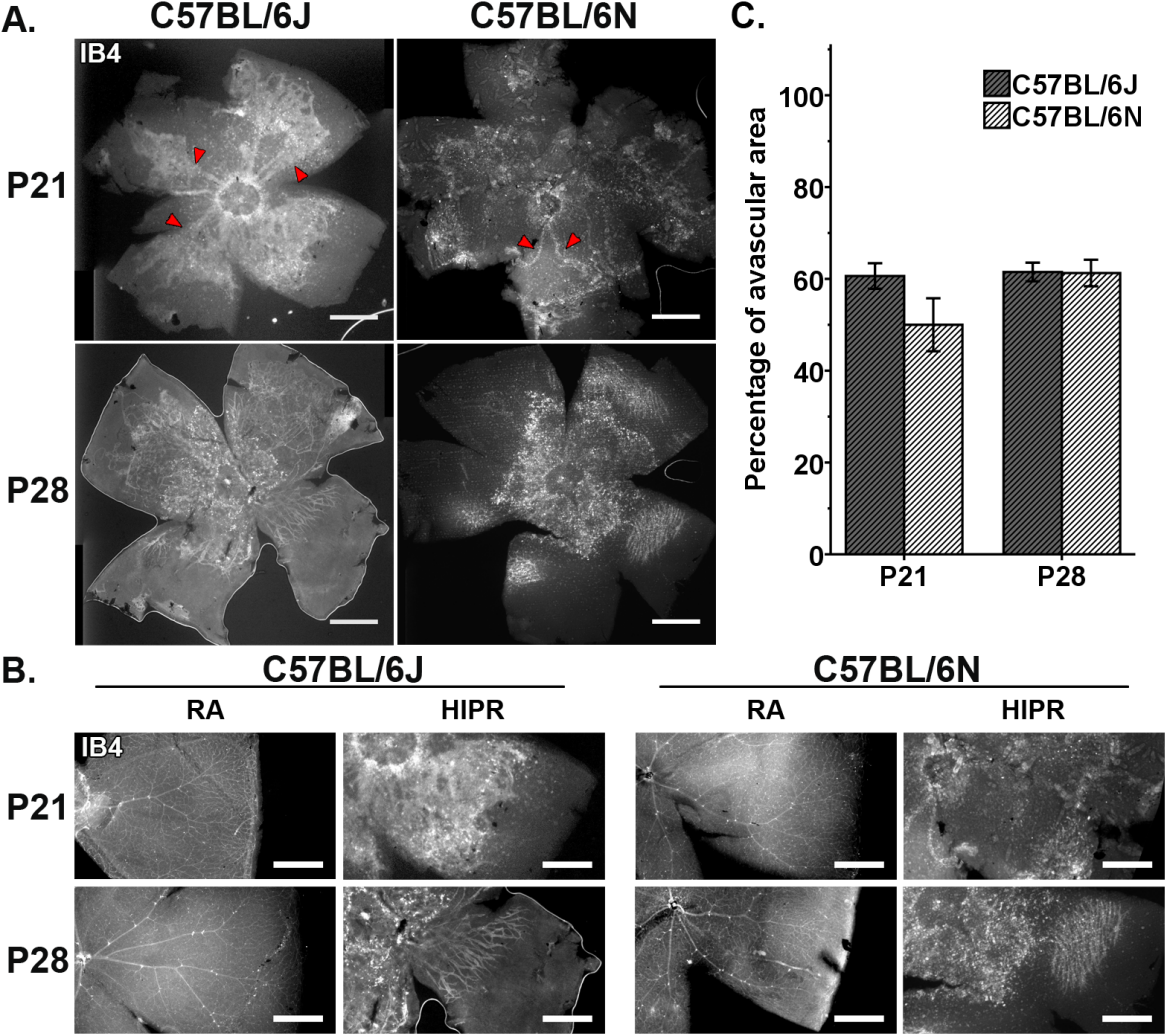
Absence of normal patterning of the retinal vasculature and disorganized angiogenesis in both strains of hyperoxia-induced proliferative retinopathy (HIPR). Representative retinal flat mounts from P21 and P28 HIPR were stained with an endothelial cell marker, IB4. (A) There is a complete absence of normal pattern retinal vasculature in HIPR of both strains. (B) Persistent hyaloid vessels (red arrowheads) were observed in the retinal mid-periphery in C57BL/6N and C57BL/6J at P21 and P28. Scale bar, 500 μm (N = 3–6). (C) There are no strain differences in the percentage of avascular area measured from the flat mounts.

We previously reported significant disruption of the retinal vascular development, with persistent hyaloidal vessels in B6J HIPR [14]. Comparing the B6J and B6N strains in HIPR, retinal flat mounts stained with IB4, an endothelial cell marker, were assessed at P21 and P28. In both strains, the retinal flat mounts revealed absent patterned retinal vasculature at P21 and P28. Instead, completely disorganized vasculature were visible surrounding the optic nerve and in the retinal mid-periphery in both strains (Fig 3). Persistent hyaloid vessels were only visible in some flat mounts (Fig 3, red arrowheads) since the hyaloidal vessels are sometimes inadvertently removed during retinal dissections. There was no qualitative or quantitative difference in these vessels comparing B6J and B6N mice (Fig 3).

### C57BL/6J and C57BL/6N hyperoxia mice manifest abnormal localization of vasculature along with Mϋller cell activation

**Fig 4:**
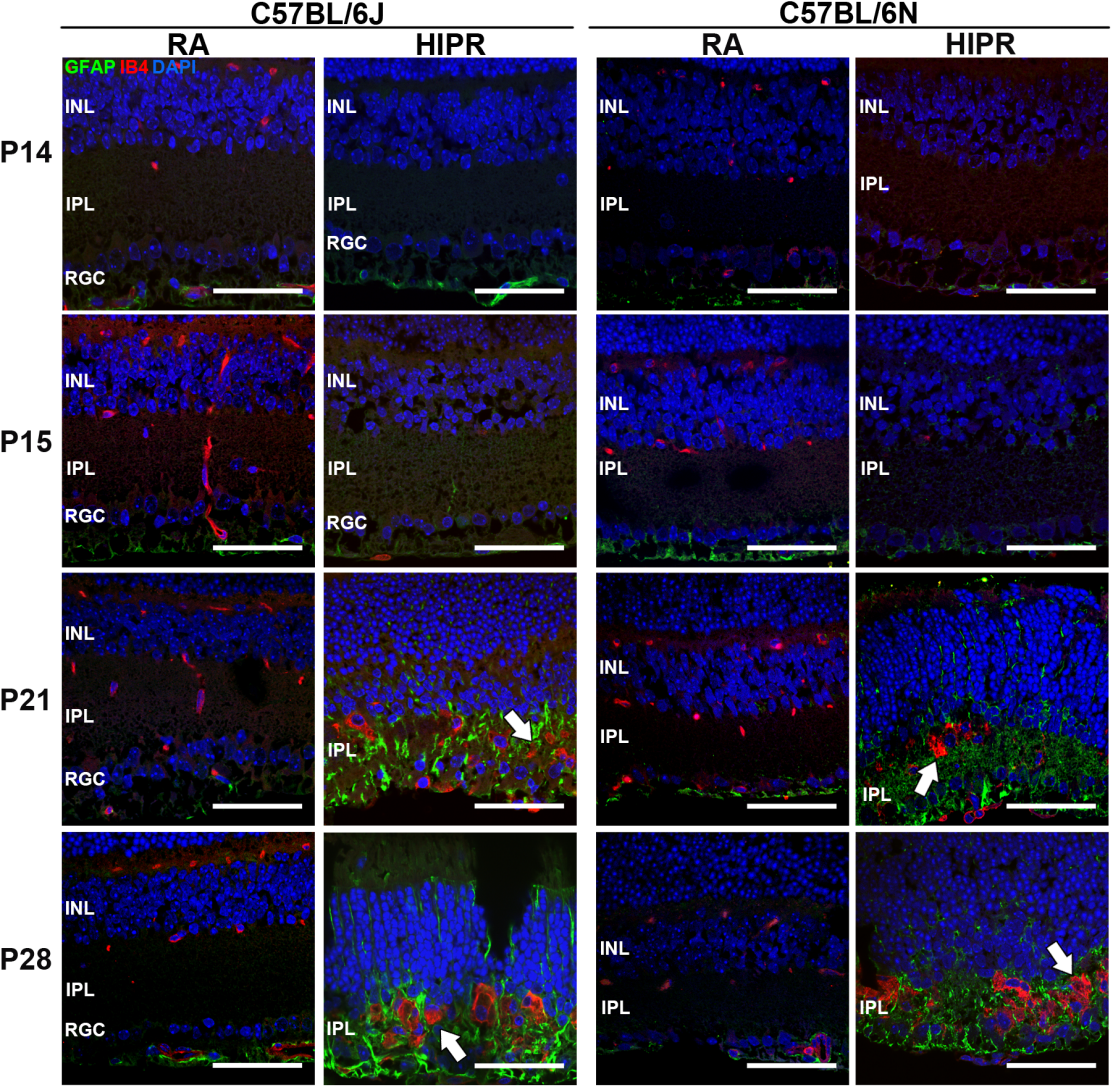
Hyperoxia-induced proliferative retinopathy (HIPR) was associated with disrupted retinal vascular development and Mϋller cell activation in both strains. Retinal cross sections were stained for endothelial cells (IB4, red), activated Mϋller cells (GFAP, green), and cell nuclei (DAPI, blue). Normal retinal capillary stratification was absent in both strains with HIPR. Instead, angiogenesis was observed in the inner plexiform layer of both strains in HIPR at P21 and P28 (white arrows). Mϋller cell activation was seen at P21 and P28 in both strains with HIPR. Representative images from the central retina are shown (N = 3). Scale bar, 50 μM. INL, inner nuclear layer. IPL, inner plexiform layer. RGC, retinal ganglion cell layer.

Based on the presence of extensive angiogenesis with failure of normal retinal vascular development (Fig 3), we wanted to evaluate the exact localization of these vessels in both strains. Retinal cross sections were stained for endothelial cells (IB4) and activated Mϋller cells (GFAP). In both strains of HIPR, there was complete absence of the retinal vasculature at P14 and P15 (Fig 4). By P21 and continuing at P28 both strains showed endothelial cell staining, appearing as vascular tufts in the inner plexiform layer (Fig 4, arrows). Activated Mϋller cells were seen in B6J and B6N HIPR mice at P21 and P28 (Fig 4). Neither strains showed any evidence of normally stratified retinal compared to room air controls.

### C57BL/6N in hyperoxia exhibit higher levels of photoreceptor reactive oxygen species compared to C57BL/6J in hyperoxia

**Fig 5:**
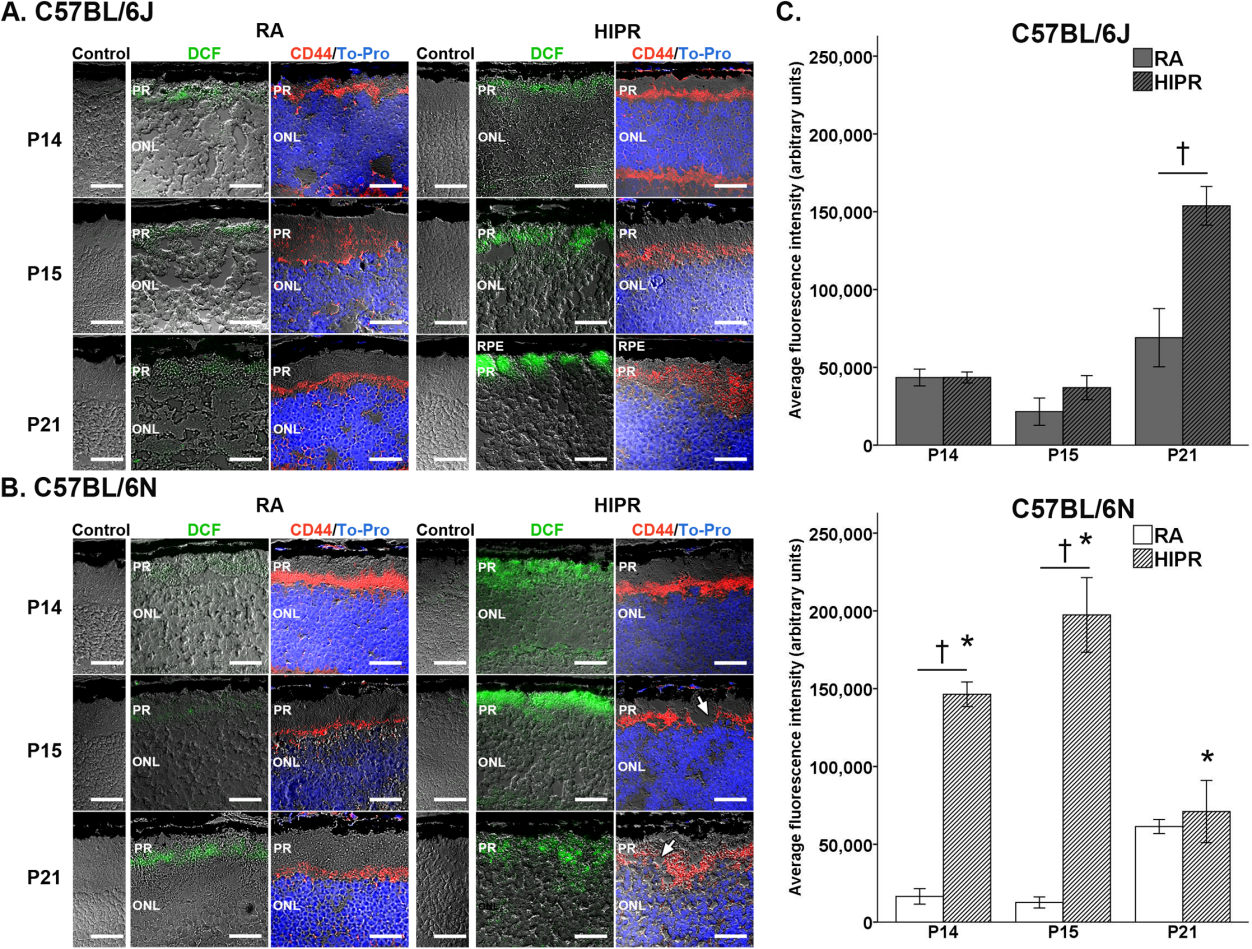
Hyperoxia-induced proliferative retinopathy (HIPR) in C57BL/6N showed increased photoreceptor reactive oxygen species compared to HIPR C57BL/6J mice. Unfixed, frozen sections from C57BL/6J (A) and C57BL/6N (B) retinas were stained with DCF stain (green) and imaged immediately. Negative control sections, with no DCF staining, were imaged at the same time. The same sections were post-fixed to stain the outer limiting membrane (CD44, red) and counterstained for cell nuclei (To-Pro, blue). (A) DCF staining in HIPR central retinas of C57BL/6J increased at P21 compared to room air (RA) controls. (B) DCF staining was elevated in HIPR central retinas of C57BL/6N at P14 and P15 compared to RA. Disruption of the outer limiting membrane was seen in C57BL/6N HIPR at P15 and P21 (arrows). Representative images are shown (N = 3). Scale bar, 25 μm. PR, photoreceptors. ONL, outer nuclear layer. RPE, retinal pigment epithelium. (C) Quantification of fluorescence intensity (N = 3 mice per group, three areas quantified from three sections per eye) was performed using corrected total cell fluorescence. Values were mean ± SEM. † p<0.05 HIPR to respective age-matched RA controls. * p<0.05 HIPR C57BL/6J compared to HIPR C57BL/6N.

We characterized oxidative stress in the retina using dichlorofluorescein (DCF) staining, which fluoresces upon contact with reactive oxygen species (ROS). In a diabetic mouse model study, DCF staining was increased in the photoreceptor inner and outer segments compared to controls [28]. Similarly, we found increased DCF staining in the photoreceptors in both strains in fresh frozen cryosections of HIPR retinas (Fig 5). Since we stained DCF on fresh frozen, unfixed tissue, we counterstained the same tissue, after fixation, with CD44, a marker for Mϋller glial end-feet for orientation and to explore whether DCF staining colocalized with OLM disruption [2, 23]. We found focal disruption of the OLM in B6N HIPR at P15 and P21 (Fig 5B, arrows), but the DCF staining was more diffuse throughout the entire photoreceptor layer (Fig 5B).

Quantification of averaged DCF corrected total cell fluorescence intensity (CTCF) showed different time-course for ROS generation in the two strains. In HIPR, B6J only showed higher DCF staining at P21 compared to controls (HIPR: 153,795 ± 12,432 AU vs control: 69,030 ± 18,702 AU, p<0.05) (Fig 5C). In contrast, B6N HIPR had significantly increased DCF staining compared to controls at P14 (HIPR: 146,414 ± 7,894 AU vs control: 16,462 ± 4,982 AU, p<0.05) and at P15 (HIPR: 197,401 ± 23,990 AU vs control: 12,591 ± 3,572 AU, p<0.05) (Fig 5C). ROS generation in B6N HIPR decreased to control levels by P21 (HIPR: 71,060 ± 20,004 AU vs controls 61,418 ± 4,506 AU) (Fig 5C).

### In hyperoxia-induced proliferative retinopathy, C57BL/6N have significantly increased NOX4 protein levels at P15 compared to C57BL/6J

NOX are a major source of ROS in the retina [29, 30]. The NOX family of proteins consists of seven isoforms (NOX 1–5, DUOX1 and DUOX2). Since NOX3 is found in the inner ear [31] and NOX5 is not present in rodents [32], we did not study them. NOX1 is located in endothelial cells and vascular smooth muscle cells [33] and is a significant source of ROS in the lung during neonatal lung hyperoxia exposure [34, 35]. When we examined NOX1 protein levels in the retina, however, we did not find any statistical differences between the strains (S2A Fig).

NOX2 and, more prominently, NOX4 are present in photoreceptors [30]. We have recently shown that NOX2 was elevated in B6J HIPR compared to controls [14]. In the current study, NOX2 protein levels were elevated in both strains (S2B Fig). The main difference in expression occurred at P21, where B6J HIPR (15.3 ± 2.2 fold change) had significantly higher NOX2 compared to B6N HIPR (3.3 ± 0.4 fold change, p<0.05, S2B Fig).

**Fig 6:**
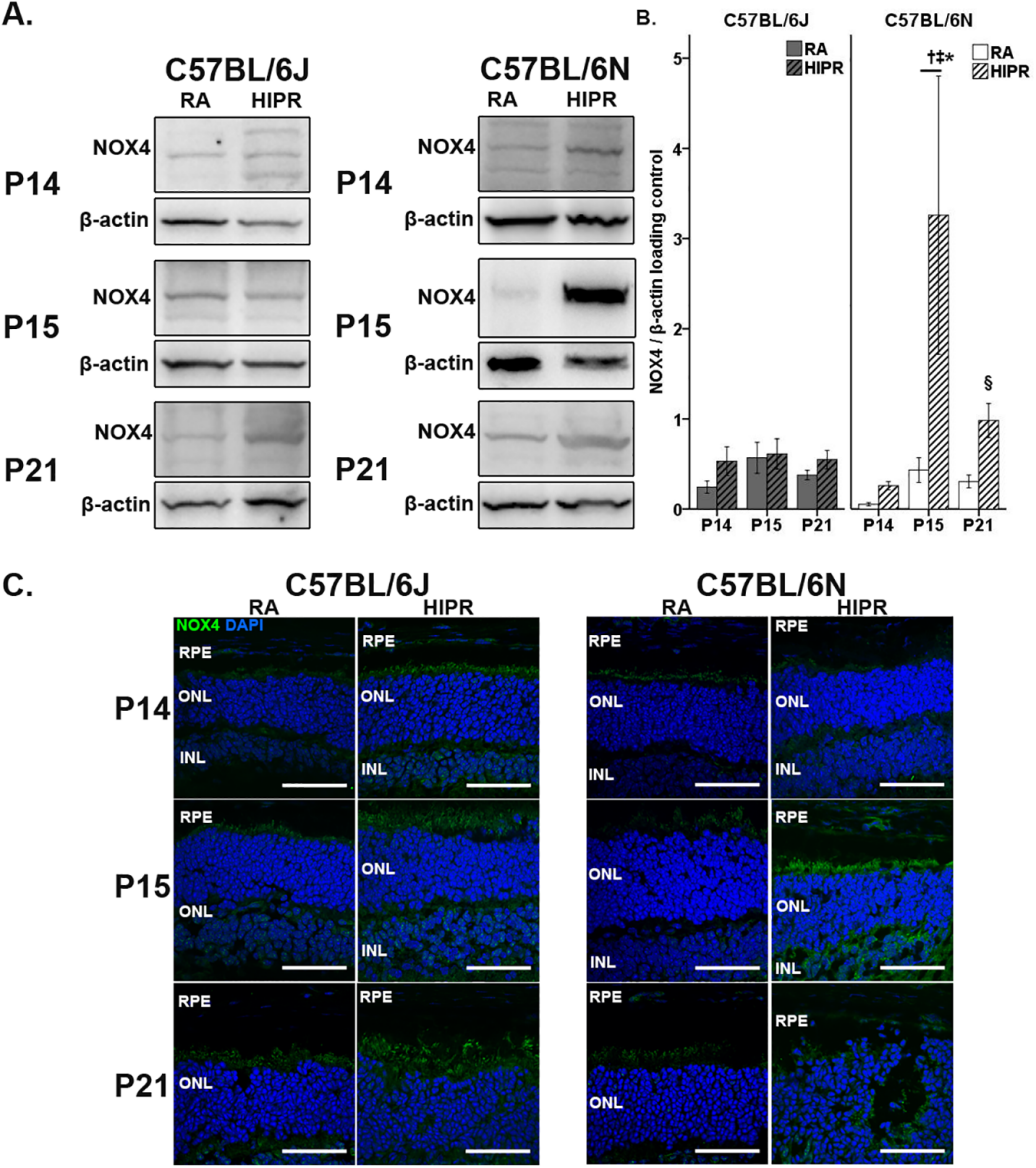
NOX4 protein expression increased in hyperoxia induced proliferative retinopathy (HIPR) in C57BL/6N compared to C57BL/6J mice. (A) Representative western blots of NOX4 (~70 kD) and β-actin (~40 kD) retinal lysates in control and HIPR mice. (B) Western blots were quantified with densitometry and normalized to a β-actin loading control. The values shown are fold-change over respective RA controls. At P15, NOX4 was significantly increased in HIPR C57BL/6N compared to HIPR C57BL/6J. * p<0.05 HIPR C57BL/6J compared to HIPR C57BL/6N. † p<0.05 HIPR to respective age-matched RA controls. § p<0.05 P21 HIPR C57BL/6N compared to P15 HIPR C57BL/6N. ‡ p<0.05 P15 HIPR C57BL/6N compared to P14 HIPR C57BL/6N. Values were mean ± SEM (N ≥ 4). (C) Retinal central cross sections reveal NOX4 was highly present in the photoreceptors in both strains (N = 3). Scale bar, 50 μm. RPE, retinal pigment epithelium. ONL, outer nuclear layer. INL, inner nuclear layer.

Since the photoreceptors were a major source of ROS in HIPR (Fig 5), we decided to determine NOX4 protein levels and their source in the retina. At all time points, we found significantly higher NOX4 levels in B6N HIPR compared to B6J HIPR (p<0.05, Fig 6A and 6B). Notably at P15, NOX4 was 19.4 ± 6.0 fold change in B6N HIPR compared to 1.1 ± 0.3 fold change in B6J HIPR (Fig 6B). The NOX4 levels in B6N HIPR decreased by P21; though they remained significantly higher than B6J HIPR (p<0.05, Fig 6B). There was stable level of NOX4 protein across the time points in B6J HIPR (Fig 6B), with a stable, low level of NOX4 in photoreceptors similar to room air controls (Fig 6C). NOX4 mainly localized in the photoreceptors in B6N HIPR (Fig 6C) and was significantly higher than B6N room air controls. In the inner retina, NOX4 localized to the blood vessels and retinal ganglion cell layer in room air controls of both strains (S3 Fig). In HIPR, NOX4 localized to the retinal ganglion cell layer in B6J, compared to more widespread staining in the inner nuclear, inner plexiform, and ganglion cell layers in B6N (S3 Fig).

### Rac1 is significantly increased at P15 in C57BL/6N in hyperoxia-induced proliferative retinopathy

**Fig 7:**
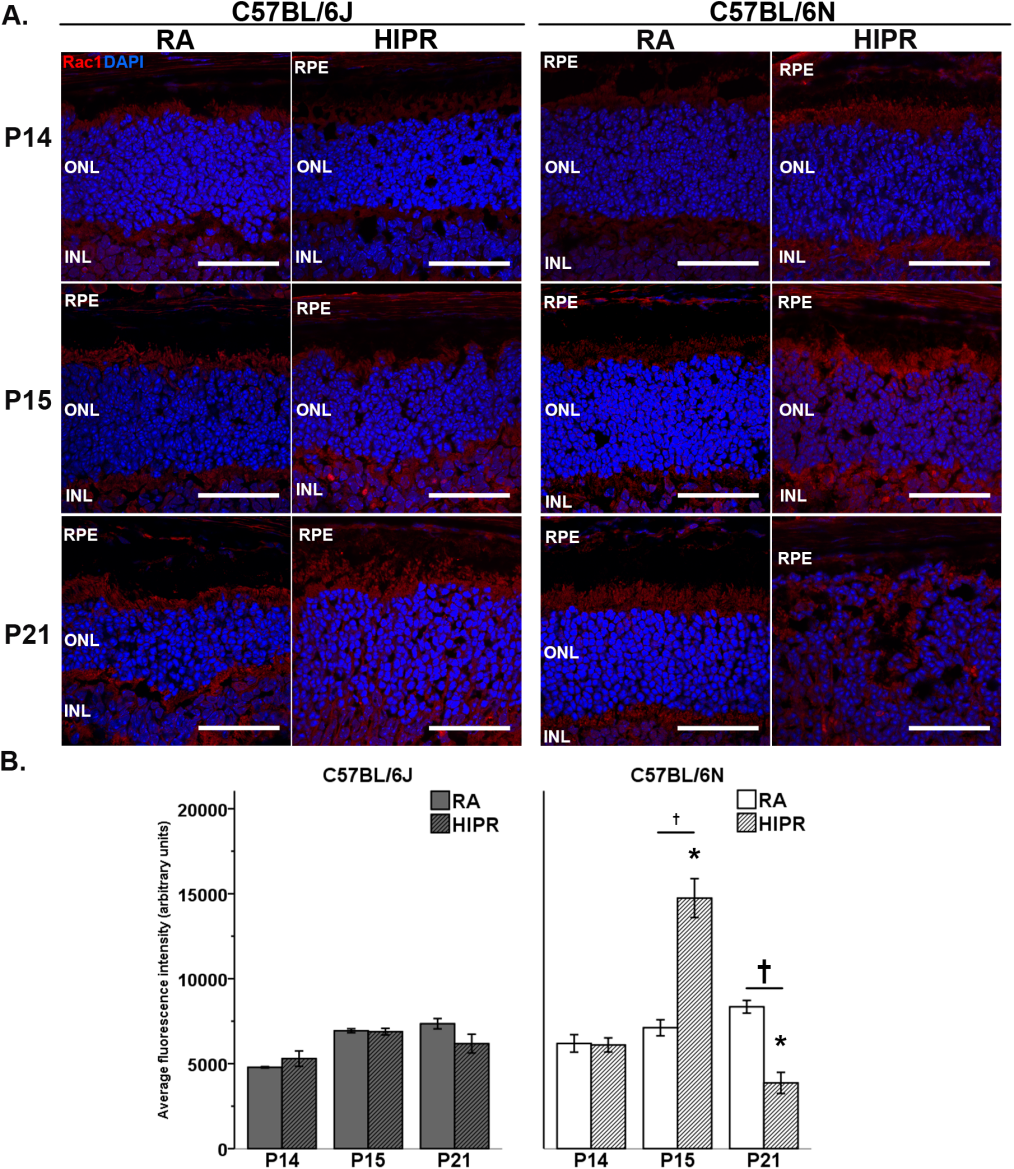
Increased Rac1 in photoreceptors of C57BL/6N P15 hyperoxia-induced proliferative retinopathy (HIPR) mice. (A) Retinal central cross sections were immunostained for the small GTPase Rac1 (red) and nuclei (blue, DAPI). C57BL/6J and C57BL/6N had Rac1 in the photoreceptors and inner retina. Representative images shown (N = 3 per group, three areas quantified from three sections per eye). Scale bar, 50 μm. RPE, retinal pigment epithelium. ONL, outer nuclear layer. INL, inner nuclear layer. (B) Quantification of fluorescence intensity in the photoreceptors using corrected total cell fluorescence is shown. C57BL/6N HIPR at P15 had increased photoreceptor Rac1. Values were mean ± SEM (N = 3). † p<0.05 HIPR to respective age-matched RA controls. * p<0.05 HIPR C57BL/6J compared to HIPR C57BL/6N.

The Rho family of GTPases includes Rac1, RhoA, and Cdc42 [36]. We sought to determine the localization of expression of all three GTPases in the retina and to examine strain differences during HIPR. We found that RhoA and Cdc42 labeled the photoreceptors, but there were no clear differences in labeling between the strains at any time point during HIPR (S5 Fig). We therefore sought to focus on Rac1, GTP-bound Rac1 was shown be increased in photoreceptors of *Drosophila* in the setting of crb knock-down [9], which we hypothesized would impact the photoreceptors in B6N as a result of the *rd8* mutation. At all time points, Rac1 was present in the photoreceptors (Fig 7A) and the inner nuclear layer (S4 Fig) in control and HIPR mice. In B6J HIPR, Rac1 staining was not significantly different compared to B6J controls (Fig 7). In contrast, B6N HIPR showed increased Rac1 at P15 in the photoreceptors and the inner nuclear layer compared to B6N room air controls (14,740 ± 1,147 vs 7,113 ± 473 AU, p<0.05) (Fig 7B). By P21 in B6N HIPR, Rac1 levels significantly decreased compared to controls (3,869 ± 620 AU vs 8,349 ± 373 AU, p<0.05, Fig 7B). This decrease in Rac1 most likely correlates with the statistically significant loss in photoreceptors at P21 in B6N due to accelerated *rd8* phenotype (Fig 1).

## Discussion

**Fig 8:**
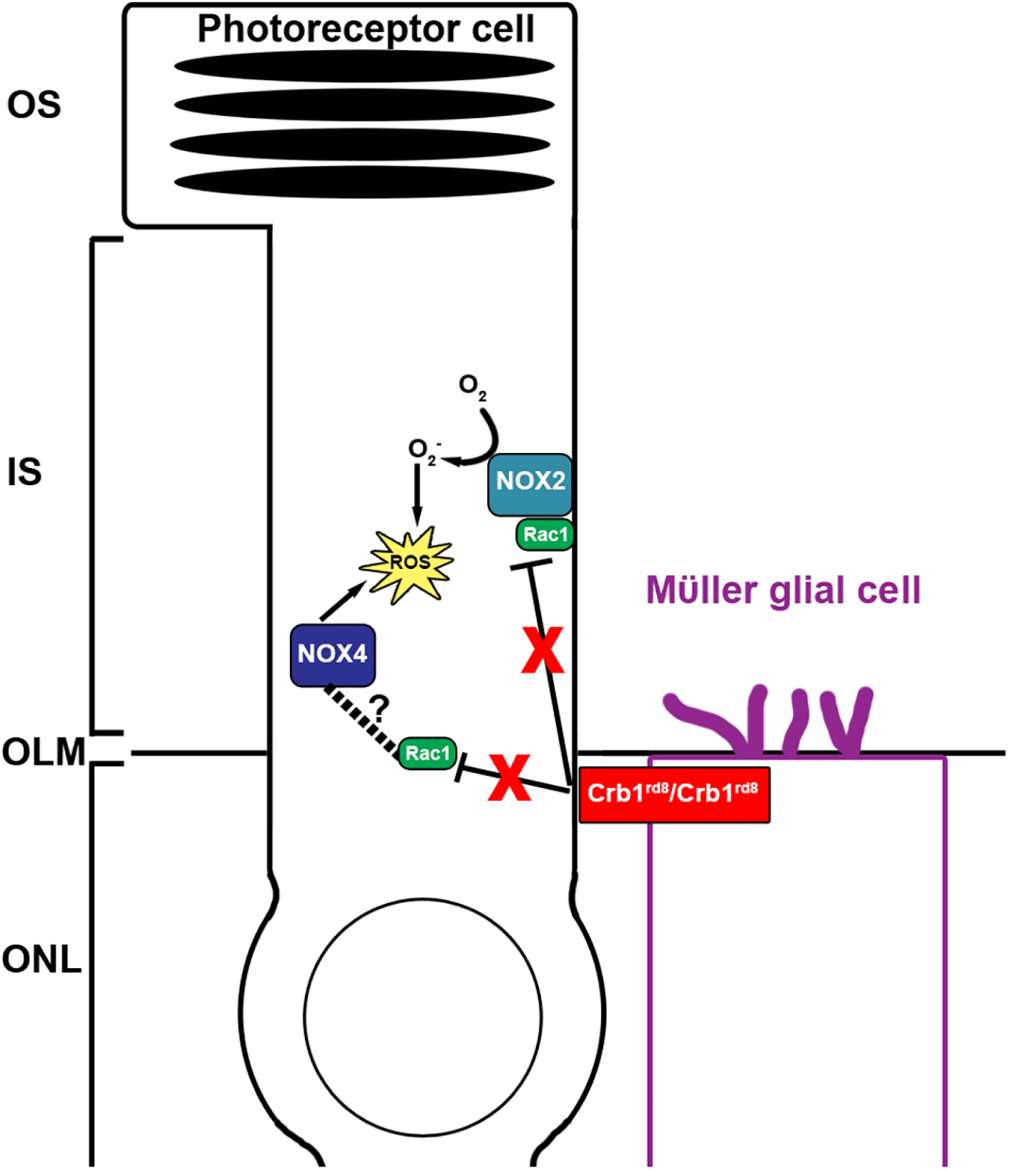
Crb1 in hyperoxia-induced proliferative retinopathy (HIPR) model. Crb1, located at the subapical region of the outer limiting membrane on Mϋller cells, connects Mϋller cells to photoreceptors. With the *rd8* mutation, Crb1 no longer regulates (inhibits) Rac1. Uninhibited Rac1 then activates NOX2 to produce superoxide. Superoxide is reactive oxygen species (ROS). It is unknown what mechanism regulates NOX4. We hypothesize (dotted line) that in photoreceptors, NOX4 is activated by a Rac1-dependent signaling mechanism. In turn, with loss of Crb1 regulation of Rac1, an increase in NOX4 produces more ROS, leading to photoreceptor damage via oxidative stress.

Our data provide evidence that neonatal oxidative stress exacerbates rosette formation in B6N mice, which we hypothesize may be related to Rac1 dysregulation. The B6N mice in HIPR showed dramatically accelerated *rd8* phenotype with severe retinal disorganization as early as P28. This finding stands in stark contrast to the slow progression of the crb1 mutation in room air raised mice, where the photoreceptor rosettes are not prevalent or functionally significant until around 35 months of age [2]. As early as P15, B6N mice undergoing hyperoxia exposure in HIPR experienced significantly increased oxidative stress with increased ROS (Fig 5), NOX4 (Fig 6), and Rac1 (Fig 7) compared to B6J strain under similar hyperoxia conditions. We hypothesize that the increase in oxidative stress in the B6N strain is due to loss of *Crb1* function leading to Rac1 dysregulation, which in turn leads to increased ROS formation and higher NOX4 expression (Fig 8).

Photoreceptors are largely susceptible to oxidative stress because of their high metabolic demand [37]. In *Drosophila*, crb knock-down showed increased ROS in the photoreceptors compared to controls, which required the presence of Rac1 and NADPH oxidase [9]. When loss-of-function crb mutant *Drosophila* were treated with apocynin, they experienced improved photoreceptor cell survival [9]. In the setting of diabetic retinopathy, another model of retinal vascular ischemia, photoreceptors have been shown to be an early source of oxidative stress [28]. Taken together, we believe the addition of hyperoxia exposure on the background of Crb1 accelerated the photoreceptor degeneration (Figs 1 and 2A) and increased NOX in the photoreceptors (Fig 6). While increased DCF staining was seen in both strains (Fig 5), NOX4 was significantly increased in the B6N strain (Fig 6), suggesting its potential role in this setting.

To understand the mechanism of accelerated rosette formation in HIPR, we explored the expression of downstream molecular targets of *Crb1*, focusing on Rac1. Rac1 has been shown to play an important role in photoreceptor morphogenesis and survival under stress. In rhodopsin-null *Drosophila*, the addition of activated Rac1 rescued photoreceptor morphogenesis [38]. In mice, Rac1 was involved in light-induced photoreceptor apoptosis [39, 40]. Conditional photoreceptor Rac1 knockout protected the retina from light-induced degeneration [40]. In turn, constitutively active Rac1 in rods lead to severe compromise in photoreceptor polarity and migration, with ultimate photoreceptor apoptosis due to NOX-induced ROS [12, 13]. In HIPR, we show that Rac1 was significantly increased at P15 in the B6N strain, localizing to the photoreceptors by immunohistochemistry, which suggests a role for Rac1-mediated acceleration of the *rd8*-related photoreceptor degeneration in the HIPR model. This finding stood in contrast to the B6J HIPR, where Rac1 levels in the photoreceptors were similar to room air controls at all time points, consistent with functional wild-type *Crb1* regulating Rac1 (Fig 7).

Since Rac1 is a member of a highly conserved family of Rho GTPases, which also include RhoA and Cdc42 [36], we wanted to explore whether the other GTPases were affected in the HIPR phenotype. However, we did not find a clear difference in photoreceptor labeling of RhoA or Cdc42 in either strain under HIPR at all time points (S5 Fig). Crb1 forms a complex that helps maintain apical polarity at the subapical adherens junction, which includes Cdc42 [41]. In a rod-specific knockdown of Cdc42 in mice with *rd1 and rd10*, Cdc42 accumulated in the perinuclear region of the photoreceptors but was not found to play a major role in photoreceptor death [42]. However, neuronal knockdown of Cdc42 during development caused loss of the outer limiting membrane in the peripheral retina and progressive retinal degeneration [43]. In a porcine retinal detachment model, RhoA activity increased within hours after the detachment [44]. Little is known about the mechanism of RhoA activation in the injured retina. Based on our immunostaining, we found that RhoA and Cdc42 were increased in the Mϋller cells of both strains at P21 under HIPR (S5 Fig). This would suggest that RhoA and Cdc42 increase in Mϋller cells, whereas Rac1 is more increased in the photoreceptors during HIPR. However, additional studies are needed to further elucidate the specific role of the different GTPases during retinal oxidative stress in other models of retinopathy, as well as in HIPR.

We explored the relative role of the different NOX and found that, similar to other retinal ischemia models [12, 45], in the B6N strain but not in B6J, NOX4 contributed to the ROS generation in photoreceptors under HIPR (Fig 6). While NOX2 requires Rac1 for activation, we found increased Rac1 expression only in B6N HIPR mice at P15 (Fig 7). Since we use whole retinal lysates for western blotting, we suspect that the increased NOX2 that we see in both strains may originate from cell types other than the photoreceptors. In a light damage mouse model, NOX2 and NOX4 were responsible for photoreceptor ROS production [45]. In a live retinal explant system, NOX4 was the most abundant NOX in photoreceptors and, with the addition of apocynin, the levels of ROS in photoreceptors were reduced [30]. While the other NOXs (NOX1, 2, 3) require Rac1, NOX4 is thought to not require Rac1 in most cells [46]. However, in mesangial cells, Rac1 was found to stimulate NOX4 ROS production [47]. The mechanism of NOX4 regulation has not been completely resolved in many cell types, including the photoreceptors [48]. Based on our findings, we suspect that NOX4 activation in the photoreceptors is Rac1-dependent, similar to mesangial cells [47] (Fig 8). To determine the exact mechanism of NOX4 activation, further molecular studies are needed that explore in photoreceptor cell culture whether NOX4 activation is dependent upon Rac1 signaling during oxidative stress.

To study other potential modifiers of the phenotype comparing the two strains, we explored inflammatory markers and Mϋller cell activation. In a study comparing the B6N and B6J strains at 3 months of age, researchers found an increase in inflammatory markers in B6N mice, with no significant difference in gene expression between the two strains [49]. In contrast to these authors, we did not observe a difference in Mϋller glial cell activation (GFAP staining; Fig 4) It is important to emphasize that there are other genetic differences between the B6N and B6J mice, in addition to the *rd8* mutation, that could contribute to the differences in inflammation and response to oxidative stress. Of note, the *Nnt* gene encodes the mitochondrial surface nicotinamide nucleotide transhydrogenase protein that is responsible for reducing NADP+ and NAD+ to NADPH and NADH.[49] The B6J strain has a deletion in the *Nnt* gene, causing the protein to be absent [50, 51], leading to higher levels of hydrogen peroxide and spontaneous oxidation of NADPH [52], This would lead to potentially enhanced oxidative stress in B6J, which we did not find [51].

Rats carrying *Crb1* mutation develop spontaneous intra-retinal angiogenesis in the inner nuclear layer [53]. Based on these findings we expected strain differences in the location and extent of angiogenesis under HIPR (Figs 3 and 4). However, comparing the vascular phenotype in HIPR, we found that both strains showed similar avascular areas and intra-retinal localization of vascular staining (Figs 3 and 4). Similarly, other researchers found no difference in the laser-induced choroidal neovascularization lesion size comparing B6N and B6J [49]. The lack of difference in angiogenesis and Mϋller cell activation between the two mouse strains in HIPR is intriguing. These two strains differ by only 12 known single nucleotide polymorphisms, making their backgrounds very similar [50, 51].

In humans, mutations in the *CRB1* gene can cause significant visual impairment, leading to a severe phenotype of retinitis pigmentosa (RP) and Leber congenital amaurosis (LCA) [54–59]. *CRB1* mutations are detected in 6% of patients with RP [60, 61] and 10–13% of patients with LCA [55, 58, 62]. Patients with *CRB1* mutations manifest either severe progressive pericentral, visual field loss in adult onset RP or profound loss of vision from birth in LCA [63]. In contrast to these severe findings in human disease, visual function and electroretinography in mice with *rd8* are not significantly different from wildtype mice, even at the most advanced stages of retinopathy (> 9 months) [63]. It is not clear why the human *CRB1* phenotypes are significantly more severe than in mice. In contrast to *rd8* (Crb1 mutants), mice deficient in Crb2 showed more severe, early disorganization throughout the entire retina [64]. Crb1 and Crb2 double knockout mice show severely impaired retinal function and thickening of the retina due to increased cell proliferation [65]. However, humans with *CRB2* mutations have rarely been reported with a retinal phenotype [66]. Recently one patient with a *CRB2* mutation has been reported, presenting with reduced visual acuity, nystagmus, and irregular retinal pigmentation [67]. This data suggests that in humans, *CRB1* mutations alone can cause severe photoreceptor degeneration, while defects in multiple components of the crumbs complex may be required to generate the severe phenotype in mice. In addition, we show that enhanced photoreceptor oxidative stress, in HIPR, leads to significant acceleration of the *rd8* phenotype, which may present an opportunity to explore the severe phenotype of LCA seen in human CRB1 mutations.

In summary, our results show that the HIPR model in the B6N mouse strain exposes the photoreceptors to significant oxidative damage, with acceleration of rosette formation and significant degeneration of the photoreceptors by P28, unlike the otherwise mild natural history of this mutation. We found significant differences in ROS, NOX4, and Rac1 between the B6J and B6N substrains in HIPR. The two strains did not show significant differences in angiogenesis or Mϋller glial cell activation. These findings highlight the importance of understanding strain differences, and alerts researchers to rule out the *rd8* (and other retinal degeneration) mutations [1, 2, 16] as potential confounders in retinal experiments, especially when ischemia and oxidative stress are introduced at a young age. Future studies of HIPR with extended time points as well as functional studies will be important to further explore differences between the strains in this model of severe retinal ischemia and angiogenesis. This model may provide researchers with an opportunity to explore the severe phenotype of Crb1 mutations in the mouse, which may simulate the severe phenotype of human CRB1. The role of oxidative stress in accelerating *rd8* phenotype suggests that higher vigilance may be indicated to avoid photoreceptor oxidative stress in infants and children carrying these mutations [68]. Based on our findings, it will be important to explore whether approaches that improve Rac1 regulation could be useful to improve photoreceptor survival in *rd8* and, ultimately human CRB1-related retinal degenerations.

## Supporting information

S1 Fig Retinal folding in C57BL/6N compared to C57BL/6J in hyperoxia-induced proliferative retinopathy. Retinal cross sections were hematoxylin and eosin stained. Rosettes involving photoreceptors were seen in C57BL/6N HIPR mice at P21 and P28 (blue arrowheads). Scale bar, 500 μm (N = 3–5).
(TIF)

S2 Fig Differential expression of NOX2 but not NOX1 in C57BL/6J and C57Bl/6N hyperoxia mice. Western blots of NOX1 (A) and NOX2 (B) retinal lysates in HIPR mice were quantified with densitometry and normalized with a β-actin loading control. The values shown were fold change over respective room air controls. (A) There were no statistical differences for NOX1 protein expression. (B) NOX2 was significantly increased at P15 in C57BL/6N HIPR mice while in C57BL/6J HIPR NOX2 was significantly increased at P21. Values were mean ± SEM (N ≥ 4). ‡ p<0.05 P15 HIPR compared to P14 HIPR of the same strain. § p<0.05 P21 HIPR compared to P15 HIPR of the same strain. * p<0.05 HIPR C57BL/6J compared to HIPR C57BL/6N.
(TIF)

S3 Fig NOX4 in the inner retina is localized in retinal ganglion cells and inner nuclear layer in hyperoxia mice. Retinal central cross sections were stained with NOX4 (green) and nuclei were stained with DAPI (blue). Minimal staining of NOX4 was present in room air control (RA) retinas in both strains, localizing to the retinal ganglion cell layer and blood vessels. In HIPR, both strains showed NOX4 staining in the retinal ganglion cell, inner plexiform, and inner nuclear layers. Representative images from the central retina are shown (N = 3). Scale bar, 50 μm. INL, inner nuclear layer. GC, ganglion cell layer. IPL, inner plexiform layer.
(TIF)

S4 Fig Hyperoxia mice in both strains have increased Rac1 in the inner retina. Retinal central cross sections stained with Rac1 (red) and counterstained with DAPI (blue) showed minimal staining in room air controls (RA). Both strains had Rac1 staining in the inner nuclear layer, inner plexiform layer, and retinal ganglion cell layer. C57BL/6N had increased Rac1 staining compared to C57BL/6J. Representative images from the central retina are shown (N = 3). Scale bar, 50 μm. INL, inner nuclear layer. GC, ganglion cell layer. IPL, inner plexiform layer.
(TIF)

S5 Fig Photoreceptor expression of RhoA and Cdc42. Retinal central cross sections stained with RhoA (A, red) or Cdc42 (B, red) and counterstained with DAPI (blue). Both strains showed minimal RhoA and Cdc42 staining in the photoreceptors and inner nuclear layer in room air controls (RA) and HIPR. There were no distinct differences detected at any time point at the level of the photoreceptors. At P21 in HIPR in both strains, RhoA and Cdc42 were increased in the Mϋller cells, compared to respective room controls. Representative images from the central retina are shown (N = 3). Scale bar, 50 μm. ONL, outer nuclear layer. RPE, retinal pigment epithelium.
(TIF)

## References

[1] Chang B, Hawes NL, Hurd RE, Davisson MT, Nusinowitz S, Heckenlively JR. Retinal degeneration mutants in the mouse. Vision Res. 2002;42(4):517–25. 11853768 10.1016/s0042-6989(01)00146-8

[2] Mehalow AK, Kameya S, Smith RS, Hawes NL, Denegre JM, Young JA, et al CRB1 is essential for external limiting membrane integrity and photoreceptor morphogenesis in the mammalian retina. Hum Mol Gen. 2003;12. 10.1093/hmg/ddg232 12915475

[3] van de Pavert SA, Kantardzhieva A, Malysheva A, Meuleman J, Versteeg I, Levelt C, et al Crumbs homologue 1 is required for maintenance of photoreceptor cell polarization and adhesion during light exposure. J Cell Sci. 2004;117(18):4169–77. 15316081 10.1242/jcs.01301

[4] Gosens I, den Hollander AI, Cremers FPM, Roepman R. Composition and function of the Crumbs protein complex in the mammalian retina. Exp Eye Res. 2008;86(5):713–26. doi: 10.1016/j.exer.2008.02.005 18407265 10.1016/j.exer.2008.02.005

[5] van Rossum AG, Aartsen WM, Meuleman J, Klooster J, Malysheva A, Versteeg I, et al Pals1/Mpp5 is required for correct localization of Crb1 at the subapical region in polarized Müller glia cells. Hum Mol Gen. 2006;15(18):2659–72. doi: 10.1093/hmg/ddl194 16885194 10.1093/hmg/ddl194

[6] Jeanes A, Smutny M, Leerberg J, Yap A. Phosphatidylinositol 3′-kinase signalling supports cell height in established epithelial monolayers. J Mol Histol. 2009;40(5–6):395–405. doi: 10.1007/s10735-010-9253-y 20157769 10.1007/s10735-010-9253-y

[7] Laprise P, Chailler P, Houde M, Beaulieu J-F, Boucher M-J, Rivard N. Phosphatidylinositol 3-Kinase Controls Human Intestinal Epithelial Cell Differentiation by Promoting Adherens Junction Assembly and p38 MAPK Activation. J Biol Chem. 2002;277(10):8226–34. doi: 10.1074/jbc.M110235200 11756422 10.1074/jbc.M110235200

[8] Chartier FJ, Hardy ÉJ, Laprise P. Crumbs controls epithelial integrity by inhibiting Rac1 and PI3K. J Cell Sci. 2011;15(124):3393–8. 10.1242/jcs.092601 21984807

[9] Chartier FJ, Hardy ÉJ, Laprise P. Crumbs limits oxidase-dependent signaling to maintain epithelial integrity and prevent photoreceptor cell death. J Cell Biol. 2012;198(6):991–8. doi: 10.1083/jcb.201203083 22965909 10.1083/jcb.201203083 PMC3444775

[10] Gassama-Diagne A, Yu W, ter Beest M, Martin-Belmonte F, Kierbel A, Engel J, et al Phosphatidylinositol-3,4,5-trisphosphate regulates the formation of the basolateral plasma membrane in epithelial cells. Nat Cell Biol. 2006;8(9):963–70. http://www.nature.com/ncb/journal/v8/n9/suppinfo/ncb1461_S1.html. doi: 10.1038/ncb1461 16921364 10.1038/ncb1461

[11] Jaffe AB, Hall A. RHO GTPASES: Biochemistry and Biology. Annu Rev Cell Dev Biol. 2005;21(1):247–69. 16212495 10.1146/annurev.cellbio.21.020604.150721

[12] Song H, Vijayasarathy C, Zeng Y, Marangoni D, Bush RA, Wu Z, et al NADPH Oxidase Contributes to Photoreceptor Degeneration in Constitutively Active RAC1 Mice NADPH Oxidase in RAC1-Induced Rod Degeneration. Invest Ophth Vis Sci. 2016;57(6):2864–75. 10.1167/iovs.15-18974 PMC5113981 27233035

[13] Song H, Bush RA, Vijayasarathy C, Fariss RN, Kjellstrom S, Sieving PA. Transgenic Expression of Constitutively Active RAC1 Disrupts Mouse Rod Morphogenesis. Invest Ophth Vis Sci. 2014;55(4):2659–68. 10.1167/iovs.13-13649 PMC4001786 24651551

[14] Lajko M, Cardona HJ, Taylor JM, Shah RS, Farrow KN, Fawzi AA. Hyperoxia-Induced Proliferative Retinopathy: Early Interruption of Retinal Vascular Development with Severe and Irreversible Neurovascular Disruption. PLoS One. 2016;11(11):e0166886 doi: 10.1371/journal.pone.0166886 27861592 10.1371/journal.pone.0166886 PMC5115836

[15] Mattapallil MJ, Wawrousek EF, Chan C-C, Zhao H, Roychoudhury J, Ferguson TA, et al The Rd8 Mutation of the Crb1 Gene Is Present in Vendor Lines of C57BL/6N Mice and Embryonic Stem Cells, and Confounds Ocular Induced Mutant Phenotypes rd8 Mutation in Vendor B6 Mice and ES Cells. Invest Ophth Vis Sci. 2012;53(6):2921–7. 10.1167/iovs.12-9662 PMC3376073 22447858

[16] Chang B, Hurd R, Wang J, Nishina P. Survey of Common Eye Diseases in Laboratory Mouse Strains Common Eye Diseases in Mice. Invest Ophth Vis Sci. 2013;54(7):4974–81. 10.1167/iovs.13-12289 PMC3723375 23800770

[17] Aslam M, Baveja R, Liang OD, Fernandez-Gonzalez A, Lee C, Mitsialis SA, et al Bone marrow stromal cells attenuate lung injury in a murine model of neonatal chronic lung disease. Am J Resp Crit Care Med. 2009;180(11):1122–30. doi: 10.1164/rccm.200902-0242OC 19713447 10.1164/rccm.200902-0242OC PMC2784417

[18] Lee KJ, Berkelhamer SK, Kim GA, Taylor JM, O’Shea KM, Steinhorn RH, et al Disrupted pulmonary artery cyclic guanosine monophosphate signaling in mice with hyperoxia-induced pulmonary hypertension. Am J Resp Cell Mol. 2014;50(2):369–78. 10.1165/rcmb.2013-0118OC PMC3930949 24032519

[19] Chou JC, Rollins SD, Ye M, Batlle D, Fawzi AA. Endothelin receptor-A antagonist attenuates retinal vascular and neuroretinal pathology in diabetic mice ETAR blockade attenuates diabetic retinopathy. Invest Ophth Vis Sci. 2014;55(4):2516–25. 10.1167/iovs.13-13676 PMC4585571 24644048

[20] Distance between lines, a plugin for ImageJ2006.

[21] Soetikno BT, Yi J, Shah R, Liu W, Purta P, Zhang HF, et al Inner retinal oxygen metabolism in the 50/10 oxygen-induced retinopathy model. Sci Rep. 2015;5:16752 doi: 10.1038/srep16752 26576731 10.1038/srep16752 PMC4649746

[22] Korystov YN, Shaposhnikova VV, Korystova AF, Emel'yanov MO. Detection of Reactive Oxygen Species Induced by Radiation in Cells Using the Dichlorofluorescein Assay. J Radiat Res. 2007;168(2):226–32. 10.1667/RR0925.1 17638409

[23] Chaitin MH, Wortham HS, Brun-Zinkernagel A-M. Immunocytochemical Localization of CD44 in the Mouse Retina. Exp Eye Res. 1994;58(3):359–66. doi: 10.1006/exer.1994.1026 7513650 10.1006/exer.1994.1026

[24] Wang J, Shanmugam A, Markand S, Zorrilla E, Ganapathy V, Smith SB. Sigma 1 receptor regulates the oxidative stress response in primary retinal Müller glial cells via NRF2 signaling and system xc−, the Na+-independent glutamate–cystine exchanger. Free Radic Biol Med. 2015;86:25–36. doi: 10.1016/j.freeradbiomed.2015.04.009 25920363 10.1016/j.freeradbiomed.2015.04.009 PMC4554890

[25] Ildefonso CJ, Jaime H, Brown EE, Iwata RL, Ahmed CM, Massengill MT, et al Targeting the Nrf2 Signaling Pathway in the Retina With a Gene-Delivered Secretable and Cell-Penetrating Peptide Ocular Gene Delivery of an Nrf2-Derived Peptide. Invest Ophth Vis Sci. 2016;57(2):372–86. 10.1167/iovs.15-17703 PMC5110262 26842755

[26] Fawzi AA, Chou JC, Kim GA, Rollins SD, Taylor JM, Farrow KN. Sildenafil Attenuates Vaso-Obliteration and Neovascularization in a Mouse Model of Retinopathy of Prematurity. Invest Ophth Vis Sci. 2014;55(3):1493–501. 10.1167/iovs.13-13207 PMC4586973 24519428

[27] Bradford MM. A rapid and sensitive method for the quantitation of microgram quantities of protein utilizing the principle of protein-dye binding. Anal Biochem. 1976;72(1):248–54. 942051 10.1016/0003-2697(76)90527-3

[28] Du Y, Veenstra A, Palczewski K, Kern TS. Photoreceptor cells are major contributors to diabetes-induced oxidative stress and local inflammation in the retina. Proc Natl Acad Sci USA. 2013;110(41):16586–91. doi: 10.1073/pnas.1314575110 24067647 10.1073/pnas.1314575110 PMC3799310

[29] Usui S, Oveson BC, Lee SY, Jo Y-J, Yoshida T, Miki A, et al NADPH Oxidase Plays a Central Role in Cone Cell Death in Retinitis Pigmentosa. J Neurochem. 2009;110(3):1028–37. doi: 10.1111/j.1471-4159.2009.06195.x 19493169 10.1111/j.1471-4159.2009.06195.x PMC2833098

[30] Bhatt L, Groeger G, McDermott K, Cotter TG. Rod and cone photoreceptor cells produce ROS in response to stress in a live retinal explant system. Mol Vis. 2010;16:283–93. 20177432 PMC2825485

[31] Bánfi B, Malgrange B, Knisz J, Steger K, Dubois-Dauphin M, Krause K-H. NOX3, a Superoxide-generating NADPH Oxidase of the Inner Ear. J Biol Chem. 2004;279(44):46065–72. doi: 10.1074/jbc.M403046200 15326186 10.1074/jbc.M403046200

[32] Lambeth JD, Kawahara T, Diebold B. Regulation of Nox and Duox enzymatic activity and expression. Free Radic Biol Med. 2007;43(3):319–31. doi: 10.1016/j.freeradbiomed.2007.03.028 17602947 10.1016/j.freeradbiomed.2007.03.028 PMC1989153

[33] Dworakowski R, Alom-Ruiz SP, Shah AM. NADPH oxidase-derived reactive oxygen species in the regulation of endothelial phenotype. Pharmacol Rep. 2008;60(1):21–8. 18276982

[34] Datta A, Kim GA, Taylor JM, Gugino SF, Farrow KN, Schumacker PT, et al Mouse lung development and NOX1 induction during hyperoxia are developmentally regulated and mitochondrial ROS dependent. Am J Physiol Lung Cell Mol Physiol. 2015;309(4):L369–L77. doi: 10.1152/ajplung.00176.2014 26092998 10.1152/ajplung.00176.2014 PMC4587628

[35] Berkelhamer SK, Kim GA, Radder JE, Wedgwood S, Czech L, Steinhorn RH, et al Developmental differences in hyperoxia-induced oxidative stress and cellular responses in the murine lung. Free Radic Biol Med. 2013;61:51–60. doi: 10.1016/j.freeradbiomed.2013.03.003 23499839 10.1016/j.freeradbiomed.2013.03.003 PMC3723750

[36] Heasman SJ, Ridley AJ. Mammalian Rho GTPases: new insights into their functions from in vivo studies. Nat Rev Mol Cell Biol. 2008;9(9):690–701. doi: 10.1038/nrm2476 18719708 10.1038/nrm2476

[37] Yu D-Y, Cringle SJ. Oxygen Distribution and Consumption within the Retina in Vascularised and Avascular Retinas and in Animal Models of Retinal Disease. Prog Retin Eye Res. 2001;20(2):175–208. 11173251 10.1016/s1350-9462(00)00027-6

[38] Chang H-Y, Ready DF. Rescue of Photoreceptor Degeneration in Rhodopsin-Null Drosophila Mutants by Activated Rac1. Science. 2000;290(5498):1978–80. 11110667 10.1126/science.290.5498.1978

[39] Belmonte MnA, Santos MF, Kihara AH, Yan CYI, Hamassaki DnE. Light-Induced Photoreceptor Degeneration in the Mouse Involves Activation of the Small GTPase Rac1. Invest Ophth Vis Sci. 2006;47(3):1193–200. 10.1167/iovs.05-0446 16505058

[40] Haruta M, Bush RA, Kjellstrom S, Vijayasarathy C, Zeng Y, Le Y-Z, et al Depleting Rac1 in mouse rod photoreceptors protects them from photo-oxidative stress without affecting their structure or function. Proc Natl Acad Sci USA. 2009;106(23):9397–402. doi: 10.1073/pnas.0808940106 19470639 10.1073/pnas.0808940106 PMC2685247

[41] Alves CH, Pellissier LP, Wijnholds J. The CRB1 and adherens junction complex proteins in retinal development and maintenance. Prog Retin Eye Res. 2014;40:35–52. doi: 10.1016/j.preteyeres.2014.01.001 24508727 10.1016/j.preteyeres.2014.01.001

[42] Heynen SR, Tanimoto N, Joly S, Selliger MW, Smamardzija M, Grimm C. Retinal degeneration modulates intracellular localization of CDC42 in photoreceptors. Mol Vis. 2011;17:2934–46. 22128240 PMC3224843

[43] Heynen SR, Meneau I, Caprara C, Samardzija M, Imsand C, Levine EM, et al CDC42 Is Required for Tissue Lamination and Cell Survival in the Mouse Retina. PLOS ONE. 2013;8(1):e53806 doi: 10.1371/journal.pone.0053806 23372671 10.1371/journal.pone.0053806 PMC3553133

[44] Wang J, Zarbin M, Sugino I, Whitehead I, Townes-Anderson E. RhoA Signaling and Synaptic Damage Occur Within Hours in a Live Pig Model of CNS Injury, Retinal DetachmentRhoA Signaling and Synaptic Damage in CNS Injury. Invest Ophth Vis Sci. 2016;57(8):3892–906. 10.1167/iovs.16-19447 PMC4974026 27472075

[45] Roehlecke C, Schumann U, Ader M, Brunssen C, Bramke S, Morawietz H, et al Stress Reaction in Outer Segments of Photoreceptors after Blue Light Irradiation. PLoS ONE. 2013;8(9):e71570 doi: 10.1371/journal.pone.0071570 24039718 10.1371/journal.pone.0071570 PMC3770596

[46] Martyn KD, Frederick LM, von Loehneysen K, Dinauer MC, Knaus UG. Functional analysis of Nox4 reveals unique characteristics compared to other NADPH oxidases. Cell Signal. 2006;18(1):69–82. doi: 10.1016/j.cellsig.2005.03.023 15927447 10.1016/j.cellsig.2005.03.023

[47] Gorin Y, Ricono JM, Kim N-H, Bhandari B, Choudhury GG, Abboud HE. Nox4 mediates angiotensin II-induced activation of Akt/protein kinase B in mesangial cells. Am J Physiol Renal Physiol. 2003;285(2):F219–F29. doi: 10.1152/ajprenal.00414.2002 12842860 10.1152/ajprenal.00414.2002

[48] Hordijk PL. Regulation of NADPH Oxidases. Circ Res. 2006;98(4):453–62. doi: 10.1161/01.RES.0000204727.46710.5e 16514078 10.1161/01.RES.0000204727.46710.5e

[49] Schnabolk G, Stauffer K, O'Quinn E, Coughlin B, Kunchithapautham K, Rohrer B. A comparative analysis of C57BL/6J and 6N substrains; chemokine/cytokine expression and susceptibility to laser-induced choroidal neovascularization. Exp Eye Res. 2014;129:18–23. doi: 10.1016/j.exer.2014.10.005 25305577 10.1016/j.exer.2014.10.005 PMC5710800

[50] Mekada K, Abe K, Murakami A, Nakamura S, Nakata H, Moriwaki K, et al Genetic differences among C57BL/6 substrains. Exp Anim. 2009;58. 10.1538/expanim.58.141 19448337

[51] Simon MM, Greenaway S, White JK, Fuchs H, Gailus-Durner V, Wells S, et al A comparative phenotypic and genomic analysis of C57BL/6J and C57BL/6N mouse strains. Genome Biol. 2013;14(7):1–22. 10.1186/gb-2013-14-7-r82 PMC4053787 23902802

[52] Ronchi JA, Figueira TR, Ravagnani FG, Oliveira HCF, Vercesi AE, Castilho RF. A spontaneous mutation in the nicotinamide nucleotide transhydrogenase gene of C57BL/6J mice results in mitochondrial redox abnormalities. Free Radic Biol Med. 2013;63:446–56. doi: 10.1016/j.freeradbiomed.2013.05.049 23747984 10.1016/j.freeradbiomed.2013.05.049

[53] Zhao M, Andrieu-Soler C, Kowalczuk L, Paz Cortés M, Berdugo M, Dernigoghossian M, et al A New CRB1 Rat Mutation Links Müller Glial Cells to Retinal Telangiectasia. J Neurosci. 2015;35(15):6093–106. doi: 10.1523/JNEUROSCI.3412-14.2015 25878282 10.1523/JNEUROSCI.3412-14.2015 PMC4397606

[54] den Hollander AI, ten Brink JB, de Kok YJM, van Soest S, van den Born LI, van Driel MA, et al Mutations in a human homologue of Drosophila crumbs cause retinitis pigmentosa (RP12). Nat Genet. 1999;23(2):217–21. http://www.nature.com/ng/journal/v23/n2/suppinfo/ng1099_217_S1.html. doi: 10.1038/13848 10508521 10.1038/13848

[55] den Hollander AI, Heckenlively JR, van den Born LI, de Kok YJM, van der Velde-Visser SD, Kellner U, et al Leber Congenital Amaurosis and Retinitis Pigmentosa with Coats-like Exudative Vasculopathy Are Associated with Mutations in the Crumbs Homologue 1 (CRB1) Gene. Am J Hum Genet. 2001;69(1):198–203. 11389483 10.1086/321263 PMC1226034

[56] Gerber S, Perrault I, Hanein S, Shalev S, Zlotogora J, Barbet F, et al A novel mutation disrupting the cytoplasmic domain of CRB1 in a large consanguineous family of Palestinian origin affected with Leber congenital amaurosis. Ophthalmic Genet. 2002;23(4):225 12567265 10.1076/opge.23.4.225.13879

[57] Khaliq S, Abid A, Hameed A, Anwar K, Mohyuddin A, Azmat Z, et al Mutation screening of Pakistani families with congenital eye disorders. Exp Eye Res. 2003;76(3):343–8. 12573663 10.1016/s0014-4835(02)00304-4

[58] Lotery AJ, Jacobson SG, Fishman GA, et al Mutations in the crb1 gene cause leber congenital amaurosis. Arch Ophthalmol. 2001;119(3):415–20. 11231775 10.1001/archopht.119.3.415

[59] Henderson RH, Mackay DS, Li Z, Moradi P, Sergouniotis P, Russell-Eggitt I, et al Phenotypic variability in patients with retinal dystrophies due to mutations in CRB1. B J Ophthalmol. 2011;95(6):811–7. 10.1136/bjo.2010.186882 20956273

[60] Bernal S, Calaf M, Garcia-Hoyos M, Garcia-Sandoval B, Rosell J, Adan A, et al Study of the involvement of the RGR, CRPB1, and CRB1 genes in the pathogenesis of autosomal recessive retinitis pigmentosa. Am J Med Genet. 2003;40(7):e89. 10.1136/jmg.40.7.e89 PMC1735523 12843338

[61] den Hollander AI, Davis J, van der Velde-Visser SD, Zonneveld MN, Pierrottet CO, Koenekoop RK, et al CRB1 mutation spectrum in inherited retinal dystrophies. Hum Mutat. 2004;24(5):355–69. doi: 10.1002/humu.20093 15459956 10.1002/humu.20093

[62] Hanein S, Perrault I, Gerber S, Tanguy G, Barbet F, Ducroq D, et al Leber congenital amaurosis: Comprehensive survey of the genetic heterogeneity, refinement of the clinical definition, and genotype–phenotype correlations as a strategy for molecular diagnosis. Hum Mutat. 2004;23(4):306–17. doi: 10.1002/humu.20010 15024725 10.1002/humu.20010

[63] Aleman TS, Cideciyan AV, Aguirre GK, Huang WC, Mullins CL, Roman AJ, et al Human CRB1-Associated Retinal Degeneration: Comparison with the rd8 Crb1-Mutant Mouse Model. Invest Ophth Vis Sci. 2011;52(9):6898–910. 10.1167/iovs.11-7701 PMC3176016 21757580

[64] Alves CH, Sanz Sanz A, Park B, Pellissier LP, Tanimoto N, Beck SC, et al Loss of CRB2 in the mouse retina mimics human retinitis pigmentosa due to mutations in the CRB1 gene. Hum Mol Gen. 2012. 10.1093/hmg/dds398 23001562

[65] Pellissier LP, Alves CH, Quinn PM, Vos RM, Tanimoto N, Lundvig DMS, et al Targeted Ablation of *Crb1* and *Crb2* in Retinal Progenitor Cells Mimics Leber Congenital Amaurosis. PLoS Genet. 2013;9(12):e1003976 doi: 10.1371/journal.pgen.1003976 24339791 10.1371/journal.pgen.1003976 PMC3854796

[66] Slavotinek AM. The Family of Crumbs Genes and Human Disease. Mol Syndromol. 2016;7(5):274–81. doi: 10.1159/000448109 27867342 10.1159/000448109 PMC5109986

[67] Lamont RE, Tan W-H, Innes AM, Parboosingh JS, Schneidman-Duhovny D, Rajkovic A, et al Expansion of phenotype and genotypic data in CRB2-related syndrome. Eur J Hum Genet. 2016;24(10):1436–44. doi: 10.1038/ejhg.2016.24 27004616 10.1038/ejhg.2016.24 PMC5027675

[68] Stone J, Maslim J, Fawzi AA, Lancaster P, Heckenlively JR. The role of perinatal stress in simplex retinitis pigmentosa: evidence from surveys in Australia and the United States. Canadian Journal of Ophthalmology / Journal Canadien d'Ophtalmologie. 2001;36(6):315–22. 11714117 10.1016/s0008-4182(01)80118-6

