# Supplementary figures and images for: Photoreceptor oxidative stress in hyperoxia-induced proliferative retinopathy accelerates rd8 degeneration
Source: PLoS One. 2017 Jul 3;12(7):e0180384. doi: 10.1371/journal.pone.0180384 (PMC5495396; doi:10.1371/journal.pone.0180384)

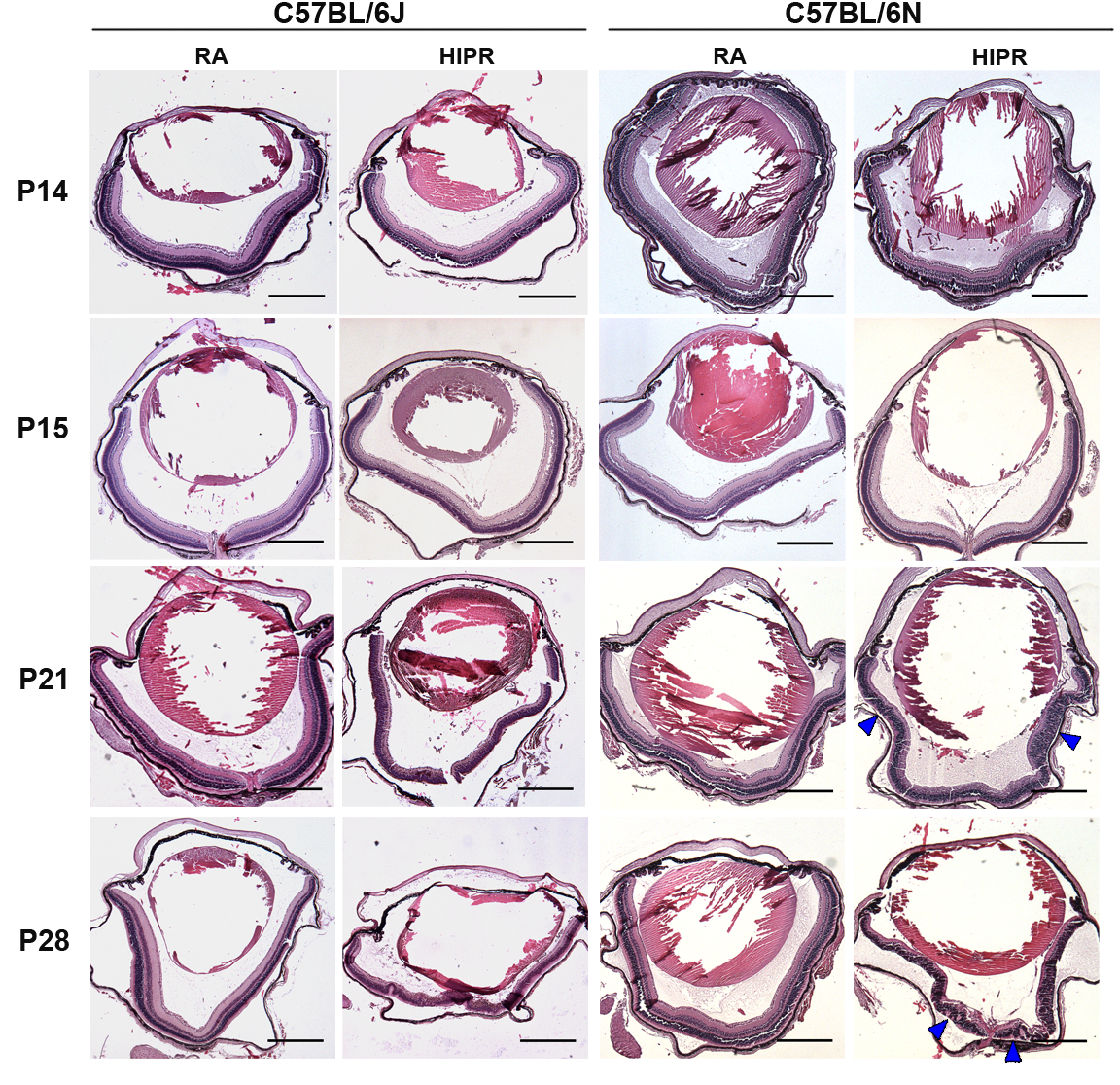

Supplement: S1 Fig — Retinal cross sections were hematoxylin and eosin stained. Rosettes involving photoreceptors were seen in C57BL/6N HIPR mice at P21 and P28 (blue arrowheads). Scale bar, 500 μm (N = 3–5). (TIF) [file pone.0180384.s001.tif]

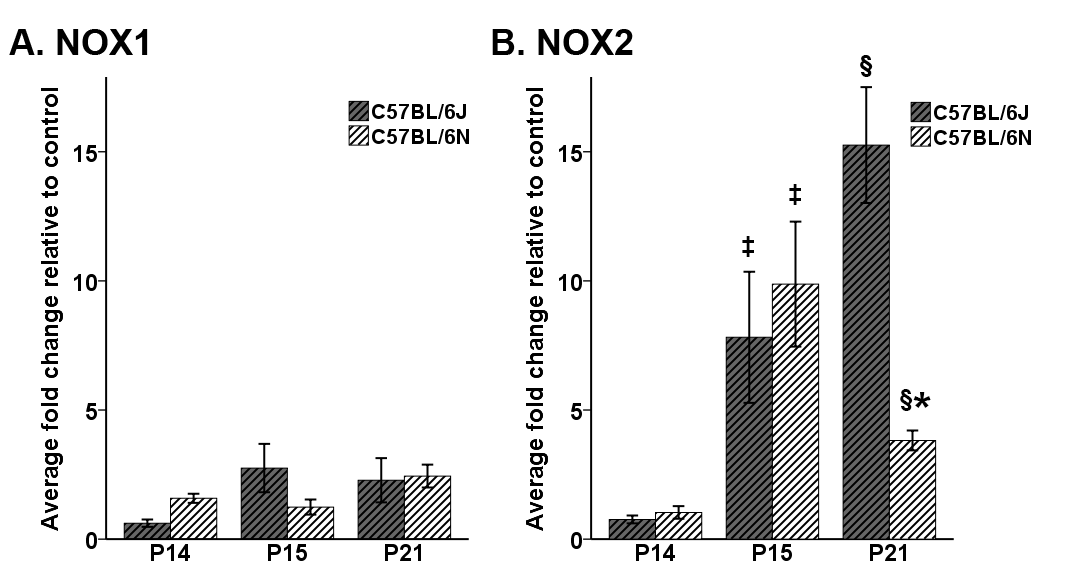

Supplement: S2 Fig — Western blots of NOX1 (A) and NOX2 (B) retinal lysates in HIPR mice were quantified with densitometry and normalized with a β-actin loading control. The values shown were fold change over respective room air controls. (A) There were no statistical differences for NOX1 protein expression. (B) NOX2 was significantly increased at P15 in C57BL/6N HIPR mice while in C57BL/6J HIPR NOX2 was significantly increased at P21. Values were mean ± SEM (N ≥ 4). ‡ p<0.05 P15 HIPR compared to P14 HIPR of the same strain. § p<0.05 P21 HIPR compared to P15 HIPR of the same strain. * p<0.05 HIPR C57BL/6J compared to HIPR C57BL/6N. (TIF) [file pone.0180384.s002.tif]

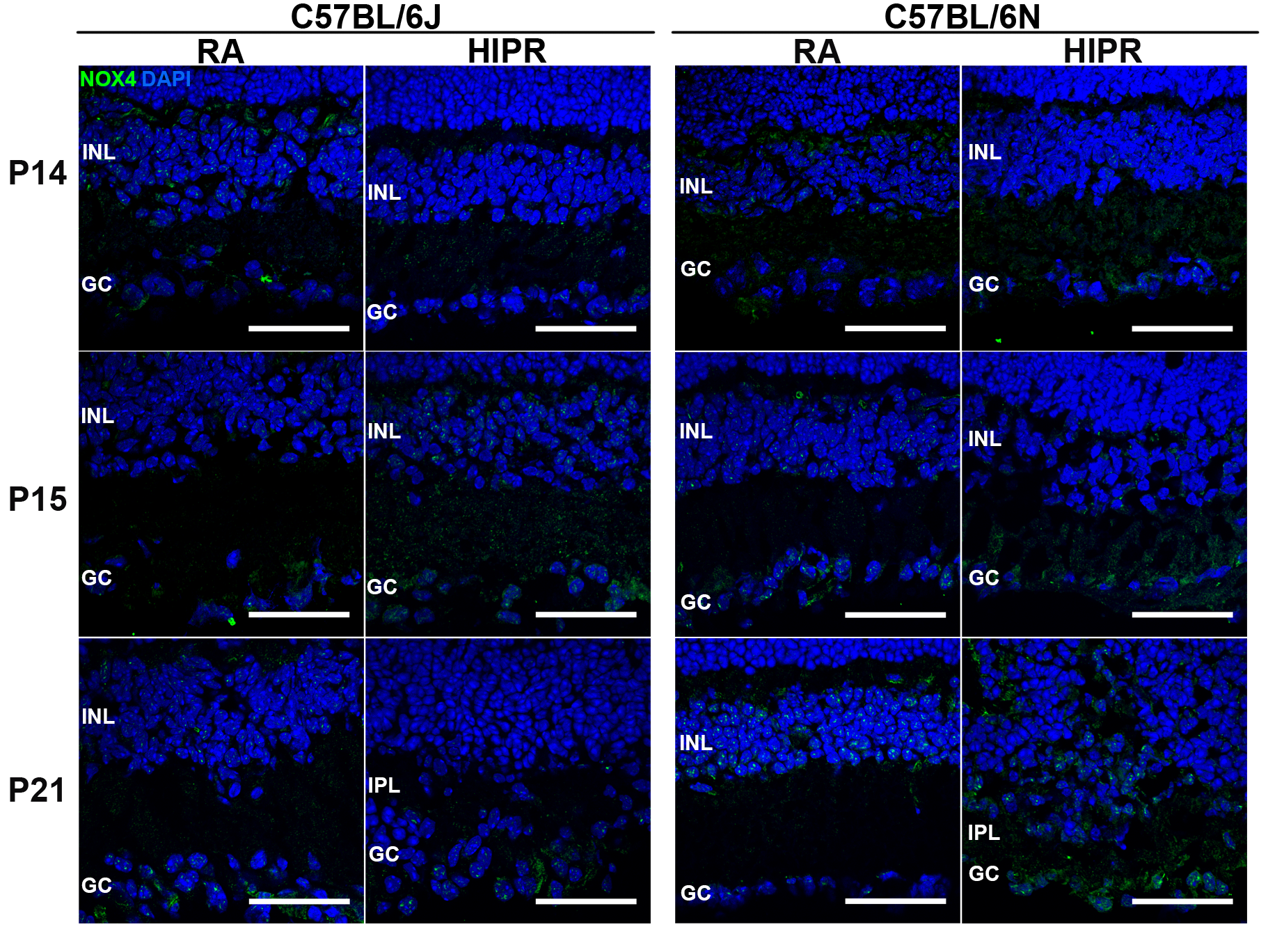

Supplement: S3 Fig — Retinal central cross sections were stained with NOX4 (green) and nuclei were stained with DAPI (blue). Minimal staining of NOX4 was present in room air control (RA) retinas in both strains, localizing to the retinal ganglion cell layer and blood vessels. In HIPR, both strains showed NOX4 staining in the retinal ganglion cell, inner plexiform, and inner nuclear layers. Representative images from the central retina are shown (N = 3). Scale bar, 50 μm. INL, inner nuclear layer. GC, ganglion cell layer. IPL, inner plexiform layer. (TIF) [file pone.0180384.s003.tif]

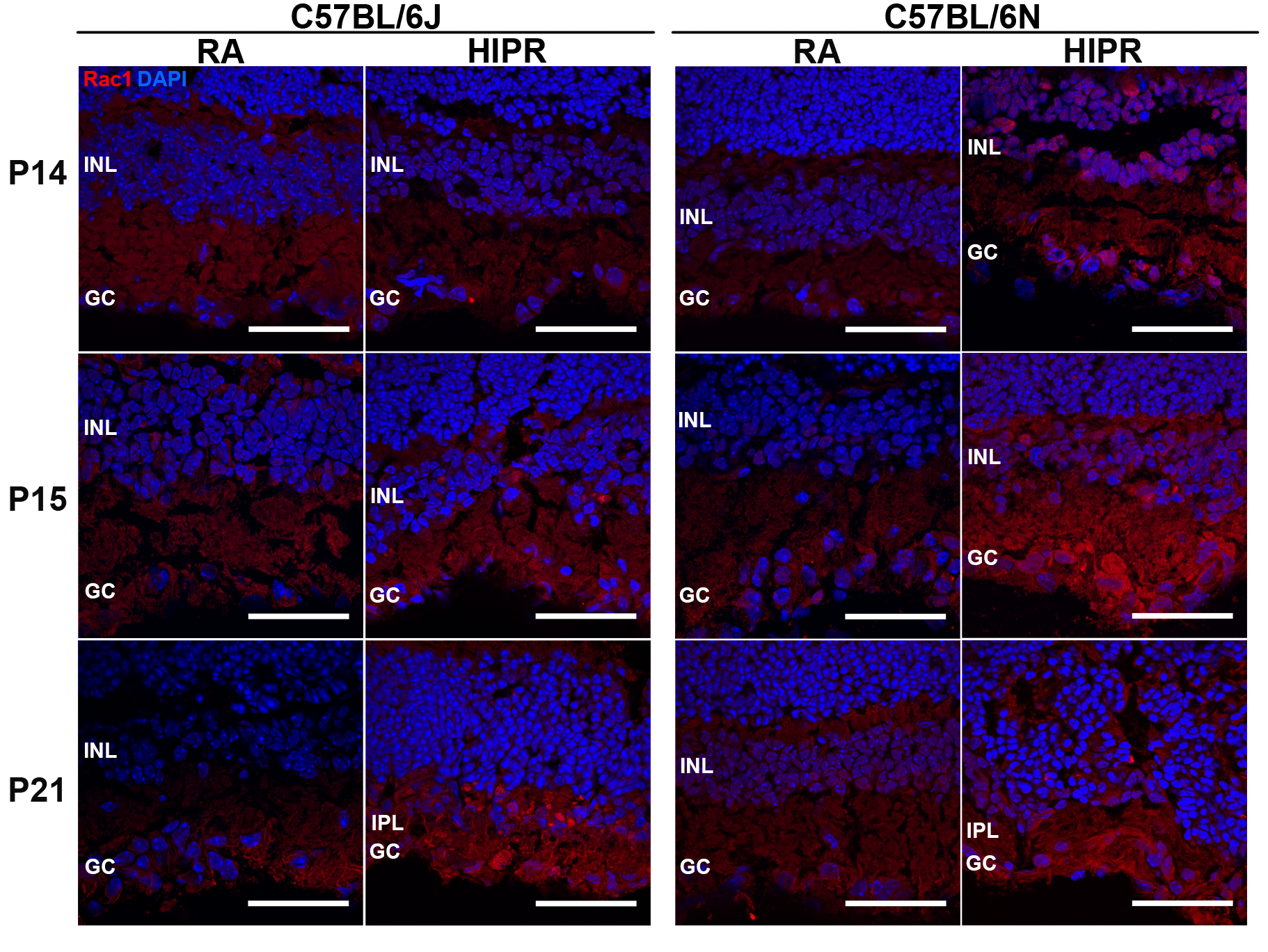

Supplement: S4 Fig — Retinal central cross sections stained with Rac1 (red) and counterstained with DAPI (blue) showed minimal staining in room air controls (RA). Both strains had Rac1 staining in the inner nuclear layer, inner plexiform layer, and retinal ganglion cell layer. C57BL/6N had increased Rac1 staining compared to C57BL/6J. Representative images from the central retina are shown (N = 3). Scale bar, 50 μm. INL, inner nuclear layer. GC, ganglion cell layer. IPL, inner plexiform layer. (TIF) [file pone.0180384.s004.tif]

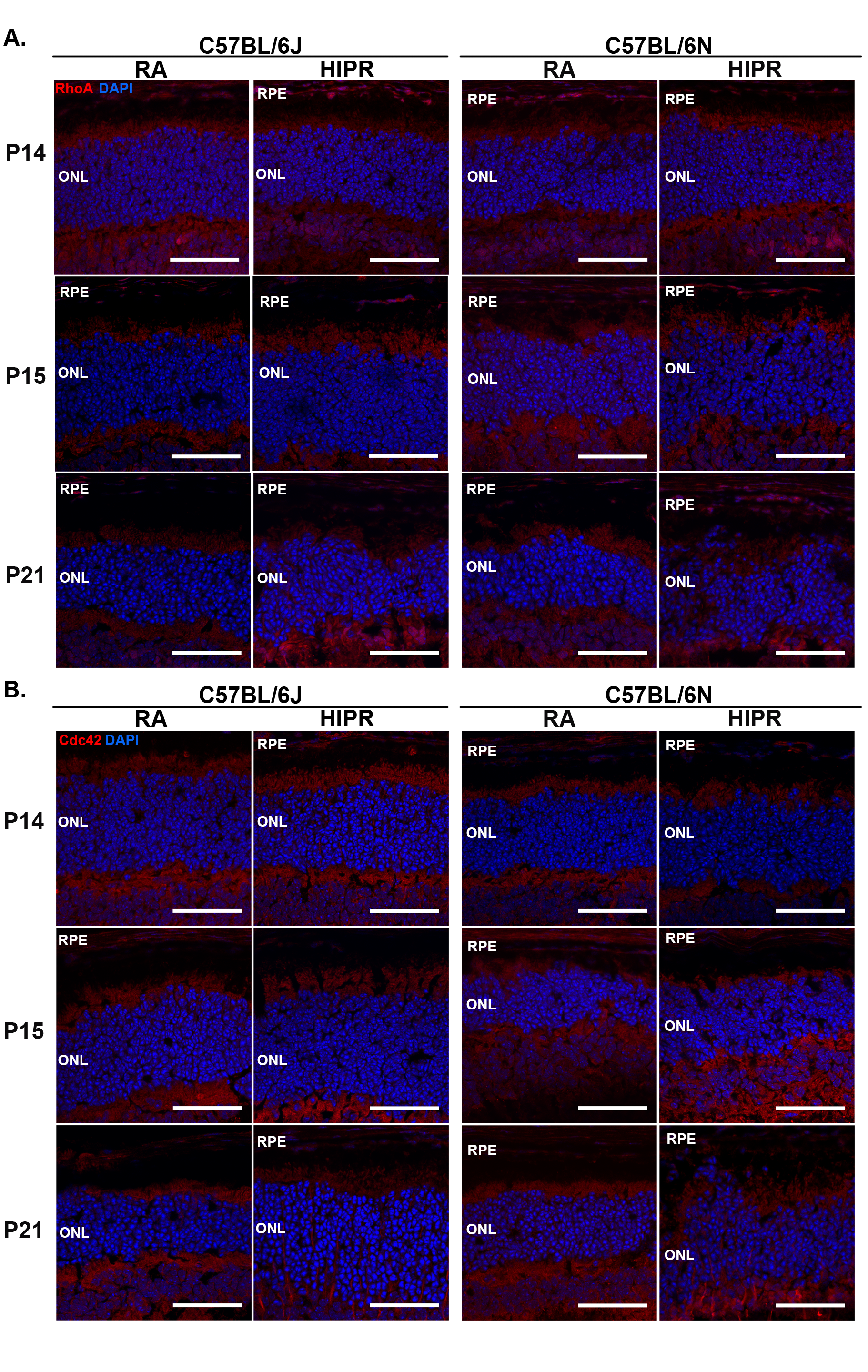

Supplement: S5 Fig — Retinal central cross sections stained with RhoA (A, red) or Cdc42 (B, red) and counterstained with DAPI (blue). Both strains showed minimal RhoA and Cdc42 staining in the photoreceptors and inner nuclear layer in room air controls (RA) and HIPR. There were no distinct differences detected at any time point at the level of the photoreceptors. At P21 in HIPR in both strains, RhoA and Cdc42 were increased in the Mϋller cells, compared to respective room controls. Representative images from the central retina are shown (N = 3). Scale bar, 50 μm. ONL, outer nuclear layer. RPE, retinal pigment epithelium. (TIF) [file pone.0180384.s005.tif]
